# Gut microbiota metabolites: potential therapeutic targets for Alzheimer’s disease?

**DOI:** 10.3389/fphar.2024.1459655

**Published:** 2024-09-17

**Authors:** Shanshan Zhang, Jing Lu, Ziqi Jin, Hanying Xu, Dongmei Zhang, Jianan Chen, Jian Wang

**Affiliations:** ^1^ The School to Changchun University of Chinese Medicine, Changchun, China; ^2^ Research Center of Traditional Chinese Medicine, The Affiliated Hospital to Changchun University of Chinese Medicine, Changchun, China; ^3^ Department of Encephalopathy, The Affiliated Hospital to Changchun University of Chinese Medicine, Changchun, China

**Keywords:** AD and gut microbiota metabolites-drug targets gut microbial metabolites, Alzheimer’s disease, microbiome-gut-brain axis, metabolites, treatments

## Abstract

**Background:**

Alzheimer’s disease (AD) is a neurodegenerative disease characterized by progressive decline in cognitive function, which significantly increases pain and social burden. However, few therapeutic interventions are effective in preventing or mitigating the progression of AD. An increasing number of recent studies support the hypothesis that the gut microbiome and its metabolites may be associated with upstream regulators of AD pathology.

**Methods:**

In this review, we comprehensively explore the potential mechanisms and currently available interventions targeting the microbiome for the improvement of AD. Our discussion is structured around modern research advancements in AD, the bidirectional communication between the gut and brain, the multi-target regulatory effects of microbial metabolites on AD, and therapeutic strategies aimed at modulating gut microbiota to manage AD.

**Results:**

The gut microbiota plays a crucial role in the pathogenesis of AD through continuous bidirectional communication via the microbiota-gut-brain axis. Among these, microbial metabolites such as lipids, amino acids, bile acids and neurotransmitters, especially sphingolipids and phospholipids, may serve as central components of the gut-brain axis, regulating AD-related pathogenic mechanisms including β-amyloid metabolism, Tau protein phosphorylation, and neuroinflammation. Additionally, interventions such as probiotic administration, fecal microbiota transplantation, and antibiotic use have also provided evidence supporting the association between gut microbiota and AD. At the same time, we propose an innovative strategy for treating AD: a healthy lifestyle combined with targeted probiotics and other potential therapeutic interventions, aiming to restore intestinal ecology and microbiota balance.

**Conclusion:**

Despite previous efforts, the molecular mechanisms by which gut microbes act on AD have yet to be fully described. However, intestinal microorganisms may become an essential target for connecting the gut-brain axis and improving the symptoms of AD. At the same time, it requires joint exploration by multiple centers and multiple disciplines.

## 1 Introduction

Alzheimer’s disease (AD) is a severe neurodegenerative disorder and the most prevalent type of dementia. It is known for causing gradual memory decline, cognitive and behavioral impairments, and sleep rhythm disturbances (Knopman et al., 2021). These symptoms significantly deteriorate the quality of life for patients and impose substantial societal burdens. According to statistics, in 2023, approximately 6.7 million individuals aged 65 and older in the United States were diagnosed with AD (Alzheimer’s disease facts and figures, 2023; Rajan et al., 2021). It is projected that by 2060, this number will increase to 13.8 million, making it the fifth leading cause of death in the country (Alzheimer’s disease facts and figures, 2023). Additionally, it is estimated that by 2050, the global number of dementia patients will reach 152 million (GBD 2019 Dementia Forecasting Collaborators, 2022). Despite the fact that the pathogenesis of AD is not completely understood, several factors influencing its progression have been identified. Among these factors, there has been a growing focus in recent years on the gut microbiome and its relationship with the brain.

The gut microbiome consists of a complex community of microorganisms residing in the gastrointestinal ecosystem, containing bacteria, fungi and viruses (Brown et al., 2023; Valdes et al., 2018). The gut microbiome, often referred to as the “second brain,” plays a role in digestion, absorption, and immune regulation while also participating in the gut-brain axis activities that influence cognition and memory. The gut microbiome communicate with the brain through neural, immune, endocrine, and metabolic pathways (Morais et al., 2021), collectively known as the microbiome-gut-brain axis (MGBA) (Zhou et al., 2024). Increasing evidence suggests that the gut microbiome play an important role in the development and progression of AD (Chen et al., 2024). Intestinal flora imbalance may occur in the prodromal stage of AD, suggesting that identifying biomarkers for mild cognitive impairment could facilitate early detection and timely treatment of AD (Li et al., 2019). Does the gut microbiome change during subjective cognitive decline (pre-dementia)?

A cohort study found a significant reduction in the abundance of the anti-inflammatory genus *Faecalibacterium* in the gut microbiota of patients with subjective cognitive decline, which was correlated with positive amyloid PET results (Sheng et al., 2021). This suggests that changes in microbial diversity and abundance are related to cognitive function, consistent with the results of another study by Sheng et al. (2022). Additionally, recent research has shown that antibiotic-induced gut dysbiosis in the *Callithrix jacchus* model leads to alterations in gut-brain axis communication mediators, which influence the cognitive and social abilities of these marmosets (Hayer et al., 2024). With the advancement of high-throughput sequencing and bioinformatics, gut microbiome and their metabolites have come into focus and may become potential therapeutic targets for AD in the future (Kuhn et al., 2019; Bharti and Grimm, 2021). Therefore, further investigation into the multi-target regulatory mechanisms of gut microbial metabolites in AD and their potential as therapeutic targets is crucial for uncovering the disease’s pathogenesis and developing innovative treatment strategies.

In recent years, as research on AD has deepened, scientists have increasingly recognized the critical role of the gut microbiome in AD pathogenesis. Based on this understanding, this review will first summarize the recent advancements in AD research, then analyze the specific mechanisms of bidirectional communication in the gut-brain axis, particularly the multifaceted regulatory roles of gut metabolites in AD, and finally explore innovative therapeutic strategies targeting gut microbiota for AD management.

## 2 Modern research on Alzheimer’s disease

**FIGURE 1:**
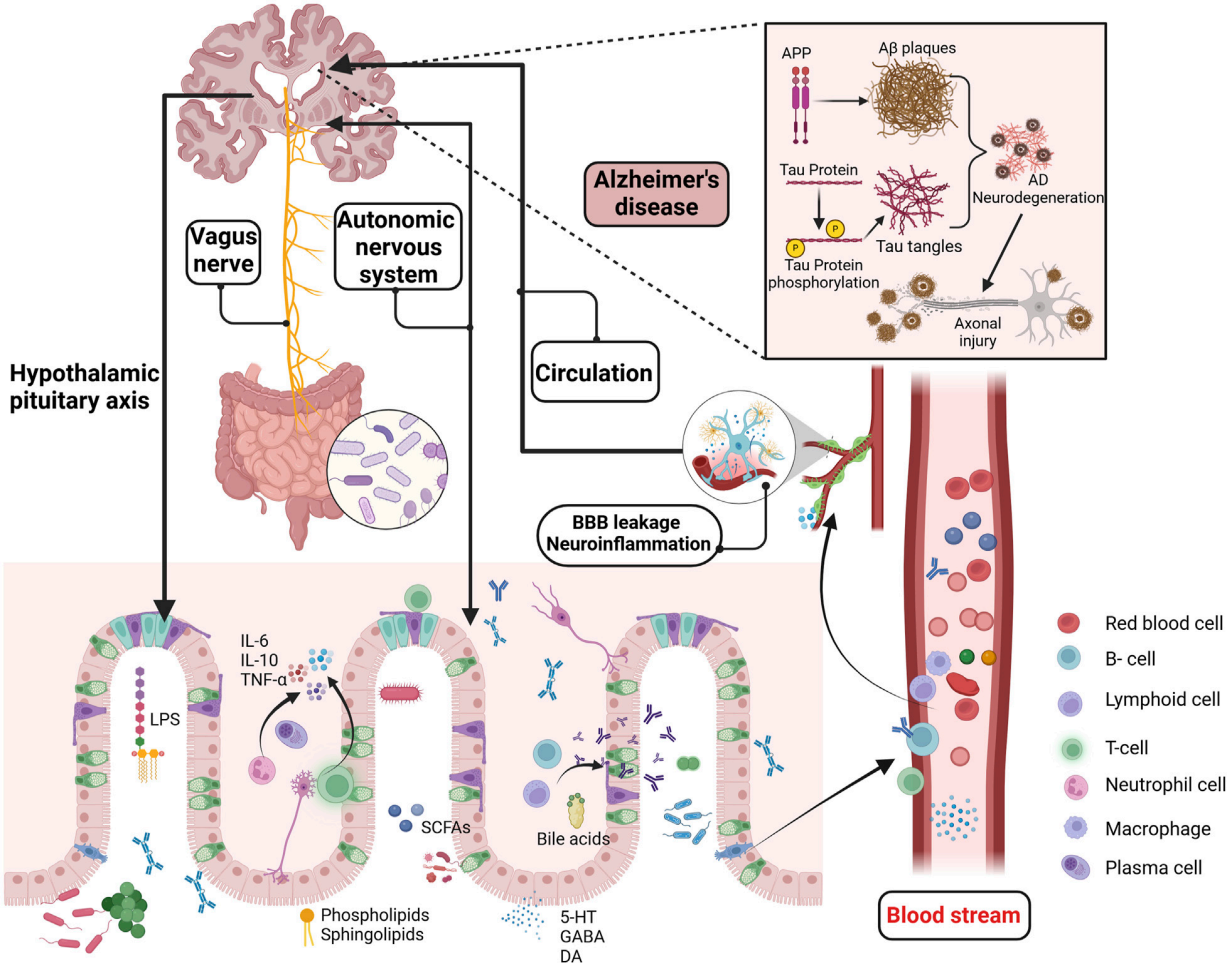
Potential mechanisms of the GMBA in AD. The GMBA facilitates bidirectional communication between the brain and gut, encompassing immune, neural, endocrine, and metabolic pathways. The microbiome produce various bioactive compounds, including sphingolipids, phospholipids, LPS, SCFAs, amino acids, BAs, GABA, and 5-HT. These compounds, released into the circulatory system or directly stimulating the nervous system (enteric, vagus nerves), can affect brain functions and potentially interfere with pathological processes like Aβ aggregation, Tau hyperphosphorylation, BBB permeability, and neuroinflammation. Moreover, the brain regulates gut functions through the HPA axis, influencing conditions like inflammatory bowel disease. GMBA, Microbiota-gut-brain axis; LPS, Lipopolysaccharide; SCFAs, short-chain fatty acids; BAs, Bile acids; GABA, γ-aminobutyric acid; 5-HT, 5-hydroxytryptamine; HPA, The hypothalamic-pituitary-adrenal; Aβ, beta amyloid. Created with (biorender.com).

AD is an age-related neurodegenerative disorder with both hereditary (Sierksma et al., 2020) and sporadic forms (Smirnov et al., 2021). It has been clearly established as a multifactorial geriatric disease (Chen and Holtzman, 2022). However, the exact pathogenic mechanisms remain to be fully elucidated. Two prevailing hypotheses have propelled AD research to new heights. The “amyloid cascade hypothesis,” proposed by Hardy and Selkoe (2002), posits that β-amyloid (Aβ) plaques in the nervous system are a primary risk factor for AD, As described in Figure 1. The accumulation of neurotoxic Aβ42 peptides in the brain is a key factor in forming amyloid plaques (Hampel et al., 2021). It is known that Aβ aggregation can trigger a neurotoxic cascade, leading to cytoskeletal alterations, neuronal dysfunction, and cell death (Chen et al., 2017). However, the precise mechanisms of Aβ′s action remain incompletely understood. Research indicated that the Toll-like receptor 2-Myeloid differentiation primary response 88 (MyD88) pathway plays a significant role in amyloid-beta formation (Rangasamy et al., 2018). It has shown that MyD88 deficiency increases the clearance of Aβ mediated by low-density lipoprotein receptor-related protein 1 (Quan et al., 2021), reducing pro-inflammatory factors and brain amyloid-beta formation (Jong Huat et al., 2023), thereby improving cognitive function in rats. However, some reports have questioned the role of MyD88, suggesting that MyD88-mediated signal transduction may not be essential for the activation of neuroglial cells and the development of brain Aβ pathology (Weitz et al., 2014).

Hyperphosphorylation of tau protein leading to neurofibrillary tangles is another significant hypothesis in the etiology of AD (Chang et al., 2021). Tau protein facilitates microtubule assembly and stability (Vershinin et al., 2007; Younas et al., 2023), and its hyperphosphorylation accelerates synaptic plasticity, coordinating memory pathways (Robbins et al., 2021). Consequently, the hyperphosphorylation of tau protein directly triggers the initiation of neurodegenerative processes associated with AD. Professor Gan Li’s (Udeochu et al., 2023) team discovered that pathogenic Tau protein induce the release of mitochondrial DNA from microglia, activating the antiviral cGAS-IFN pathway and leading to sustained IFN release. This impairs synaptic integrity and plasticity, resulting in persistent cognitive dysfunction (Udeochu et al., 2023). Recent studies suggested that phosphorylated tau protein 217 could serve as a reliable biomarker for AD (Mattsson-Carlgren et al., 2024), detectable with ultra-sensitive graphene field-effect transistor sensors (Wang et al., 2024). This advancement significantly enhanced early screening and diagnosis of AD. Researchers have proposed the “dual prion disorder” hypothesis, which suggests that the interaction between Aβ and tau proteins could be a target for intervention (Aoyagi et al., 2019; Gomes et al., 2019). Studies have shown that the interplay between Aβ and tau pathologies leads to neuronal loss and synaptic damage (Samura et al., 2006; Shin et al., 2007), resulting in memory decline in AD (Ribé et al., 2005). These findings elucidate why monotherapies targeting Aβ or tau alone have failed to achieve satisfactory outcomes in clinical treatments (Lewis et al., 2001).

Beyond the mainstream hypotheses of Aβ cascade and abnormal tau protein phosphorylation (Ju and Tam, 2022), there are prominent theories regarding neuroinflammation (Self and Holtzman, 2023), mitochondrial dysfunction (Calvo-Rodriguez et al., 2024), and cholinergic hypotheses (Chen et al., 2023a), with the scientific community still actively investigating the pathogenesis of AD. Interestingly, an increasing number of studies have identified chronic inflammation from periodontitis as a potential risk factor for AD (Sparks Stein et al., 2012). Specifically, infection with *Treponema* spp. has been associated with an increased incidence of AD (Nemergut et al., 2022), possibly due to the activation of neuroinflammation in the hippocampus following alveolar bone resorption, which subsequently promotes Tau hyperphosphorylation in mice (Tang et al., 2022). Additionally, AβPP is a significant component of *Treponema* spp. biofilms and senile plaques, and it is crucial in the increased amyloid plaque deposition observed in periodontitis patients (Miklossy, 2016). The close connection between the microbiota and the central nervous system offers new strategies for studying neurodegenerative diseases. In addition to the peripheral inflammation-induced AD development, such as from periodontitis, the role of the MGBA in AD and other neurodegenerative diseases has also garnered significant attention. Previous reports have dentified elevated levels of Aβ in the intestines of both humans and mice, with Aβ42 increasing with age in the intestines of APP/PS1 mice (Jin et al., 2023). Additionally, isotope tracing and fluorescent labeling have shown that intestinal Aβ42 is primarily transported to the brain via the bloodstream, effectively activating microglial cells (Jin et al., 2023). Consequently, the gut may serve as a significant source of cerebral Aβ, representing an important mechanism in the pathogenesis of AD (Ferreiro et al., 2023), as described in Figure 1.

Extensive research has demonstrated that gut microbial metabolites, as key regulators within the MGBA, influence AD-related pathological processes through various pathways. Studies suggested that the gut microbiome communicates with microglial cells via the secretion of metabolites and neurotransmitters (Wardman et al., 2022). *Bacteroides fragilis*, a commensal gut microbe, produce metabolites such as 12-Hydroxyheptadecatrienoic acid and Prostaglandin E2 that activate microglial cells in neuronal C/EBPβ transgenic mice, inducing the onset of AD. This process is associated with the activation of the C/EBPβ/Asparaginyl endopeptidase pathway and the enrichment of polyunsaturated fatty acids (Xia et al., 2023). Additionally, short-chain fatty acids (SCFAs) are significant signaling molecules derived from microbial metabolism and play an important role in maintaining blood-brain barrier (BBB) integrity and inhibiting tau protein uptake (Rauch et al., 2020). Numerous experiments and clinical researche have demonstrated the role of gut microbiome dysbiosis in cognitive impairments in hosts (Li N. et al., 2023; Ornish et al., 2024; Aljumaah et al., 2022). Recent studies have also highlighted the potential therapeutic roles of probiotics and prebiotics in modulating gut-brain interactions, particularly in the context of AD (Abdelhamid et al., 2024). *Bifidobacterium*, one of the most widely studied probiotic strains, has been shown in multiple double-blind clinical trials to modulate gut microbiota, which may contribute to slowing cognitive decline (Shi et al., 2022; Azuma et al., 2023) and inhibiting the progression of brain atrophy (Asaoka et al., 2022). Additionally, certain strains of *Lactobacillus* (Xiao-Hang et al., 2024)and *Lactobacillus plantarum* (Önning et al., 2023) have demonstrated potential in improving cognitive function and significantly reducing Aβ deposition and the hyperphosphorylation of Tau proteins in AD animal models (Nie et al., 2024; Zhang et al., 2024). Interestingly, meta-analyses have also found that probiotics improve cognitive function in AD patients by reducing levels of inflammatory and oxidative biomarkers (Den et al., 2020). Notably, prebiotics, in addition to probiotics, have also shown positive effects on cognitive function. Several clinical studies have found that a diet rich in prebiotics significantly enhances cognitive performance in patients with mild cognitive impairment, accompanied by improvements in gut microbiota diversity (Ni Lochlainn et al., 2024; Abe et al., 2024; Berding et al., 2021). In summary, gut microbiota dysbiosis regulates the pathogenesis of AD through multiple mechanisms, as shown in Figure 1.

Over the years, researchers have been trying to develop drugs in animal and clinical studies to treat AD, aiming to reduce Aβ aggregation, mitigate neurofibrillary tangles, and alleviate neuroinflammation. Unfortunately, these approaches have not succeeded due to poor efficacy and adverse events. In addition to the previously mentioned factors, mitochondrial dysfunction (Rai et al., 2020), neuroinflammation (Moore et al., 2019), autophagy defects (Pradeepkiran and Reddy, 2020), γ-aminobutyric acid (GABA) functional impairments (Bi et al., 2020), synaptic damage (Peng et al., 2022), genetics and gender are also high-risk factors for AD. Although the exact pathogenic mechanisms of AD remain unclear, the bidirectional communication mechanism of the gut-brain axis has gradually become a focal point of research among the many potential pathogenic factors.

## 3 Bidirectional communication between the gut and the brain

**FIGURE 2:**
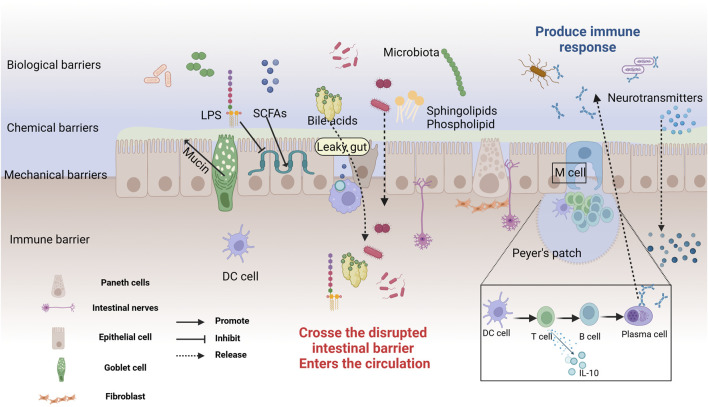
The structure and Defensive Functions of the Intestinal Barrier. The intestinal mucosal barrier consists of microbial, chemical, mechanical, and immune barriers. The microbial barrier, primarily formed by the gut microbiota, acts as the first line of defense. The chemical barrier is made up of mucus and digestive fluids secreted by intestinal epithelial cells, and antibacterial substances produced by microbes. Goblet cells secrete a translucent mucin protein distributed on the intestinal mucosal surface to prevent bacterial binding to epithelial sites. Probiotics secrete antibacterial substances such as sphingolipids, SCFAs, BAs, amino acids, and polysaccharides, working together to prevent pathogen invasion and maintain intestinal environmental homeostasis. The mechanical barrier, also known as the physical barrier, consists of various types of intestinal mucosal epithelial cells. Tight junctions between cells effectively block the invasion of bacteria and endotoxins. The immune barrier includes gut-associated lymphoid tissue and dispersed immune cells. Peyer’s patches are the sites for immune induction and activation, comprising T and B cells, which can secrete cytokines to combat external antigens. In cases of gut leakage, metabolites and neurotransmitters can cross the compromised barrier, entering the circulation and potentially triggering systemic effects. Created with (biorender.com).

The intestinal mucosa, the most extensive surface mucous membrane in the human body, serves not only as a critical site for food digestion and absorption but also as the largest microbial reservoir (Seo et al., 2021). The intestinal mucosal barrier, an innate defense maintaining gut environmental equilibrium and blocking pathogens and toxins, comprises mechanical (tightly connected intestinal epithelium), chemical (mucus and antimicrobial substances), microbial (intestinal probiotics), and immune (immune system cells) (Gao et al., 2024), as depicted in Figure 2. The mechanical barrier of the intestinal mucosa, composed of intestinal epithelial cells, tight junctions, and the mucus layer covering these cells, is essential for maintaining gut ecological balance (Xu et al., 2024). The gut microbiome plays a significant role in influencing the function of this barrier, as illustrated in Figure 2. Increasing evidence supports the bidirectional communication between the gut and the brain, where the gut microbiota plays a crucial role in maintaining gut barrier integrity and regulating neural function (Wang Y. et al., 2023; Aburto and Cryan, 2024).

While certain species, such as *Lactiplantibacillus plantarum* (Nie et al., 2024; Li et al., 2024), *Bacteroides fragilis* (Chatzidaki-Livanis et al., 2017) and *Bifidobacterium* (Cani et al., 2009), enhance the expression of tight junction proteins, others, like toxins from Lachnospiraceae spp. (Wen et al., 2022a), can compromise the integrity of the intestinal barrier. Additionally, microbial metabolites, including SCFAs and aromatic compounds, contribute to the crosstalk between the gut microbiota and the BBB, thereby influencing brain function and disease progression (Salminen, 2023). Studies have found that 27-hydroxycholesterol exacerbates cognitive impairment in AD model rats (Wang et al., 2020). This effect may be associated with the disruption of gut barrier integrity, which is influenced by gut microbiota dysbiosis. Additionally, research has shown that the enrichment of Lipopolysaccharide (LPS), cell adhesion molecules, and pro-inflammatory factors in the blood of AD patients is closely associated with gut microbiota dysbiosis (Marizzoni et al., 2023). The increased expression of brain amyloid protein and plasma pTau-181 may also be mediated by gut microbiota imbalance. Under stable conditions, the symbiotic microbiome suppresses pathogen colonization and maintains the integrity of the intestinal barrier (Seo et al., 2021). In contrast, certain pathogens or microbial metabolites produce high levels of inflammatory factors (such as TNFα), which downregulate tight junction expression and increase intestinal permeability. This allows larger molecules, like bacteria and metabolites, to pass through the intestinal barrier and reach various organs, including the brain (Schirmer et al., 2019). Goblet cells are specialized epithelial cells in the gut that secrete mucins, which are essential for forming the intestinal mucus layer and maintaining gut barrier permeability (Cai et al., 2023; Yang et al., 2022). This mucus not only prevents bacteria from adhering to the epithelium but also plays a crucial role in immune function. Research has shown that *Akkermansia muciniphila* enhances the secretion of goblet cells and mucins, which helps repair damaged gut barriers. This action further regulates the Th1/Th2 balance, reduces levels of pro-inflammatory substances like LPS, and improves spatial learning and memory deficits in AD model mice (Ou et al., 2020; He et al., 2022). Additionally, *Bifidobacterium adolescentis* regulates the Treg/Th2 balance, which relies on increased mucin production and the reshaping of the gut microbiota. This process is also crucial for reducing intestinal inflammation (Fan et al., 2021). Intriguingly, intestinal permeability tests indicated that in AD patients, tight junction proteins (such as claudins, occludin, and zonula occludens-1) expression is altered, leading to increased intestinal permeability and facilitating the entry of bacterial endotoxins into the bloodstream (André et al., 2019), which affects the central nervous system. Animal studies further corroborate that gut dysbiosis is linked to increased BBB permeability, which can be ameliorated by restoring gut microbial homeostasis (Gasaly et al., 2021). Early exposure to antibiotics or the use of probiotics increases the expression of tight junction proteins, reduces BBB permeability, and decreases Aβ accumulation and Tau protein abnormalities. These effects are likely associated with the reconstruction of gut microbiota (Seo et al., 2023; Liu et al., 2021). Additionally, the gut microbiome is implicated in the induction and recruitment of reactive astrocytes to Aβ plaques. Male APPPS1-21 mice treated with short-term broad-spectrum antibiotics showed a reduction in Aβ plaques and significant changes in astrocyte morphology (Chandra et al., 2023a).

The gut-brain axis is a communication system integrating neural, endocrine, and immune signals between the gut and the brain, serving as a crucial pathway for gut-brain interactions (Sun et al., 2020). This bidirectional system allows the brain to influence gastrointestinal physiology (such as intestinal motility and mucus secretion) and immune functions (involving immune cells and the production of stress-related factors) through the vagus nerve, autonomic nervous system, and endocrine system (Luo et al., 2024), like Figure 1. The vagus nerve serves as a key node in MGBA signal transmission, enabling the relay of gut signals to the brain via the enteric nervous system and vagus nerve (Kaczmarczyk et al., 2017). The hypothalamic-pituitary-adrenal (HPA) axis is a vital component of the neuroendocrine system and constitutes a major pathway for gut-brain communication. The HPA axis, as a crucial component of the neuroendocrine system, is also a major pathway for gut-brain communication (Zhou et al., 2024). Under stress, the hypothalamus releases corticotropin-releasing factor, activating the HPA axis and ultimately stimulating the adrenal glands to secrete cortisol (Wu et al., 2021). Cortisol can profoundly impact gut health by increasing intestinal permeability, promoting inflammation, and weakening gut barrier function (Hayer et al., 2024). When the HPA axis is overactive, disruptions in gut barrier function and increased local inflammation can negatively impact the stability of the gut microbiota and the immune response (Matenchuk et al., 2020). Additionally, the brain influences gut function by modulating immune responses. For example, under stress, neuroendocrine pathways can alter the activity of immune cells in the gut and change the composition of the gut microbiota, leading to gut dysfunction and impaired barrier integrity (Shaler et al., 2021). Recent studies have further revealed how this complex bidirectional communication influences the development of neurodegenerative diseases, particularly the imbalance in the gut-brain axis observed in AD patients (Porter et al., 2000). While the brain profoundly impacts the gut through neuroendocrine and immune pathways, conversely, the gut microbiota and its metabolites also influence brain function through various mechanisms, particularly in regulating the BBB and neuroinflammation. An imbalance in the gut microbiota is a critical factor in the development of cognitive dysfunction. The study by Aljumaah et al. (2022) found that an increased relative abundance of *Prevotella* and *Dehalobacterium* was associated with reduced cognitive function scores, which could be reversed by supplementation with the probiotic *Lactobacillus rhamnosus*. Importantly, neurotransmitters, amino acids, and other metabolites produced by the gut microbiota can communicate with the central nervous system through various pathways. Among these, the activation of the aryl hydrocarbon receptor (AhR) is believed to be closely related to the integrity of the BBB (Maciel et al., 2018). The high expression of AhR in BBB cells has been widely documented, and the activation of its signaling pathway is closely associated with increased BBB permeability and loss of integrity (Ren et al., 2021; Bobot et al., 2020). Gut-derived metabolites influence the renin-angiotensin system and nitric oxide metabolism through AhR signaling (Salminen, 2023; Eckers et al., 2016), weakening vascular dilation, leading to reduced cerebral perfusion, and ultimately impairing the BBB function, triggering neuroinflammation and neurodegenerative changes. This evidence further establishes the importance of the gut-brain axis in AD pathology, where the gut microbiota significantly influences AD pathogenesis by mediating the crosstalk between AhR and the BBB.

Increasing evidence suggests that gut microbial metabolites influence the progression of AD by regulating the host immune system and inflammatory responses through the bidirectional communication of the gut-brain axis (Sun et al., 2020). Studies have demonstrated that neurotoxic T cells play a crucial role in mediating tau aggregation and subsequent neurodegeneration (Chen et al., 2023b). CD8 T cells and Treg cells are essential for maintaining immune tolerance (Su et al., 2023). Both can limit AD pathology by inhibiting microglial activation and amyloid protein deposition, which are key to improving learning and memory functions (Reagin and Funk, 2023; Yeapuri et al., 2023). Recent evidence suggests that immune dysregulation caused by gut microbiota imbalance may be related to the development of AD (Das and Ganesh, 2023). A series of studies have found the presence of cytokines and immune cell proliferation in the peripheral systems of AD patients and animal models (Nijhuis et al., 1994; Lambracht-Washington et al., 2009). The gut microbiome may regulate peripheral immune responses by releasing cytokines, complement, and microbial metabolites (including sphingolipids and LPS). In the presence of endothelial dysfunction, these inflammatory mediators and metabolites are recruited into the central immune system, thereby exacerbating the progression of AD (Jacobs et al., 2024). Therefore, inflammation overactivation and systemic inflammatory responses induced by gut microbiota dysbiosis may be related to the activation of the peripheral and central innate immune systems (Castro-Gomez and Heneka, 2024). As the Human Microbiome Project progresses, it has become clear that changes in the gut microbiome composition and metabolites affect various body organs, including the central nervous system, through multiple pathways. This knowledge has opened new avenues for research into neurodegenerative diseases. In 2012, the concept of the MGBA was formally introduced (Cryan and Dinan, 2012), revealing the fundamental mechanism that the gastrointestinal tract and brain are closely connected through the commensal microbiome. Both endogenous and exogenous factors can alter the composition and activity of the microbiome, with the metabolites derived from the microbiome serving as key mediators in host-microbiome interactions and central bioactive substances in gut-brain communication (Gasaly et al., 2021). Metagenomic and metabolomic analyses have revealed that abnormal activities in AD model mice are associated with gut microbiota imbalances and corresponding changes in related metabolites (Sun P. et al., 2022).

Increasing evidence supported bidirectional communication and regulation between the gut and the brain via the MGBA (Morais et al., 2021; Chen et al., 2024), although the precise details of this mechanism remain unclear. The vagus nerve may be a critical node in the signaling of the MGBA pathway, facilitating the transmission of information from the gut to the brain through enteric and vagal nerves (Kaczmarczyk et al., 2017; Bravo et al., 2011). Conversely, the brain can regulate gut function via the vagus nerve. Metabolites from the microbiome activate gut immune cells (such as regulatory T cells,B cells, and group 3 innate lymphoid cells), and the resulting inflammatory factors (such as interleukin-6) enter the bloodstream through the compromised intestinal mucosal barrier, affecting the integrity of the BBB and contributing to brain diseases (Gasaly et al., 2021), as described in Figure 2. This suggests that microbial metabolites regulate host brain function in various ways. Sphingolipids, as bioactive lipids, are crucial for signal transduction processes (Brown et al., 2023). Recent studies indicated that bacterial sphingolipids can activate the immune system and trigger the release of inflammatory factors, affecting neuronal survival and mitochondrial apoptosis, thereby inducing neuroinflammation associated with brain disorders (Tan et al., 2021). Fatty acids, as energy providers for the human body, also play a significant role in immune regulation (Luo et al., 2024; Hamilton et al., 2022). SCFAs produced by microbial degradation are potential therapeutic targets for AD (Zhou Y. et al., 2023). They influence cognition and the accumulation of pathological substances in AD patients through various pathways, including mediating immune regulation, activating the HPA axis, and promoting the synthesis of neurotrophic factors (Qian et al., 2022). These metabolites maintain homeostasis and regulate brain function through direct and indirect pathways.

The impacts on host biology are typically mediated by various metabolites, involving neurotransmitters, digested carbohydrates, proteins, lipids, and bile acids (Knauf et al., 2020). The complex microbial communities in the gut shape gastrointestinal physiology and regulate systemic metabolism (Brown et al., 2023; Barreto and Gordo, 2023). Microbiome translocation can disrupt the balance of various metabolites, such as endogenous lipid synthesis (Johnson et al., 2020) and tryptophan metabolism (Wikoff et al., 2009), which in turn may affect cytokines and the immune system related to AD pathogenesis (Castro-Gomez and Heneka, 2024). These microbial products, as active mediators of the gut-brain axis, may occupy a central role in the MGBA pathway (Loh et al., 2024), representing potential targets for AD treatment.

## 4 Multi-target regulation of AD by microbial metabolites

The microbiome substantially impacts the host’s metabolomic profile. Specifically, the microbiome is a primary source of endogenous lipids (Le et al., 2022) and tryptophan metabolites (Wikoff et al., 2009), and it also plays a role in the transformation and absorption of exogenous lipids (Li Y. et al., 2022). The gut microbiome can produce a wide variety of bioactive compounds (Wardman et al., 2022). These compounds affect the central nervous system through the enteric and vagus nerves, while also regulating the intestinal barrier and the BBB, maintaining the host’s homeostasis (Kaczmarczyk et al., 2017). These compounds primarily include diet-related metabolites, endogenous molecular metabolites, and signaling molecules from microbial cell walls. Next, we will delve into the multiple mechanisms by which lipid metabolites, bile acids, amino acids, and neuroactive substances regulate the onset and progression of AD, as depicted in Table 1.

**TABLE 1:** The role of gut microbe-derived products in the pathology of AD.

Microbial product		Producers	Effect	Refs
Lipid	Sphingolipid	*Bacteroides*, *α-Proteobacteria*	Restored the intestinal mucosal barrier, regulates microglia	Johnson et al. (2020), Heaver et al. (2022), Qiu et al. (2021), Zhang et al. (2022a)
	Phospholipid	*Akkermansia muciniphila*, *Desulfovibrio*	Associated with immune system activation, mitochondrial function, and oxidative stress	Li et al. (2022a), Zappelli et al. (2022), Daniele et al. (2020), Che et al. (2018)
	LPS	*Bacteroides fragilis*, *Escherichia coli*, *Shigella flexneri*	Microglial activation mediated by TLR4/TREM2 imbalance, release of pro-inflammatory factors and Aβ accumulation	Azzam et al. (2023), Jiang et al. (2021a), Wu et al. (2017), Agostini et al. (2020)
	SCFAs	*Bacteroides*, *Firmicutes*, *Akkermansia muciniphila*	Inhibited Tau protein phosphorylation, reduced oxidative stress and release of pro-inflammatory factors, regulated microglial homeostasis	Xie et al. (2023a), Wen et al. (2020), Matt et al. (2018), Jiang et al. (2021b)
BAs		*Bifidobacteriu*, *Bacteroides*	Regulates Tau and Aβ accumulation and promotes mitochondrial biogenesis	Zangerolamo et al. (2021), Ochiai et al. (2021), Song et al. (2024), West et al. (2020), Bell et al. (2018)
Amino acid		*Clostridium sporogenes*, *Escherichia coli,* *Lactobacillus gasseri*	Harms: destroyed the cytoskeleton and activated oxidative stress Benefits: Regulates microglial polarization and reduces oxidative stress	Bakker et al. (2023), Jacobs et al. (2019), Pan et al. (2024), Noristani et al. (2012), Brymer et al. (2023)
Neurotran-smitters	GABA	*Lacticigenium*, *Bacteroides*, *Bifidobacterium*, *Escherichia coli*	Harms: promoted the spread of Aβ and Tau pathologies Benefits: promoted neuronal differentiation	Osse et al. (2023), Andersen et al. (2021), Tozuka et al. (2005), Jo et al. (2014)
	5-HT	*Lacticigenium*, *Bifidobacterium*	Increased vagus neuron activity, regulated astrocyte and microglia activity, reduced Aβ aggregation and Tau phosphorylation	Ma et al. (2023), Reddy et al. (2021), Sood et al. (2023), Labus et al. (2021)
	Acetylcholine	*Lactiplantibacillus plantarum*, *Bacillus subtilis*, *Escherichia coli*, *Staphylococcus aureus*	Promoted the deposition of Aβ amyloid plaques and induced hippocampal atrophy	Sun et al. (2022c), Zhong et al. (2022), Teipel et al. (2023)
	Dopamine	*Bacillus*, *Bacteroides*, *Brevilactibacter*, *Bifidobacterium*	Regulated the intensity of excitatory synapses in neurons, affected cognition and mood	Possemato et al. (2023), La Barbera et al. (2022), Spoleti et al. (2024)
	NE	*Escherichia coli*, *Proteus vulgaris*, *Bacillus subtilis*	Regulated synaptic plasticity, upregulate BDNF, reduced pro-inflammatory factors, increased amyloid clearance	Fukabori et al. (2020), Giustino et al. (2020), Cao et al. (2021)

### 4.1 Lipid metabolites and gut microbiome

#### 4.1.1 Sphingolipid

**FIGURE 3:**
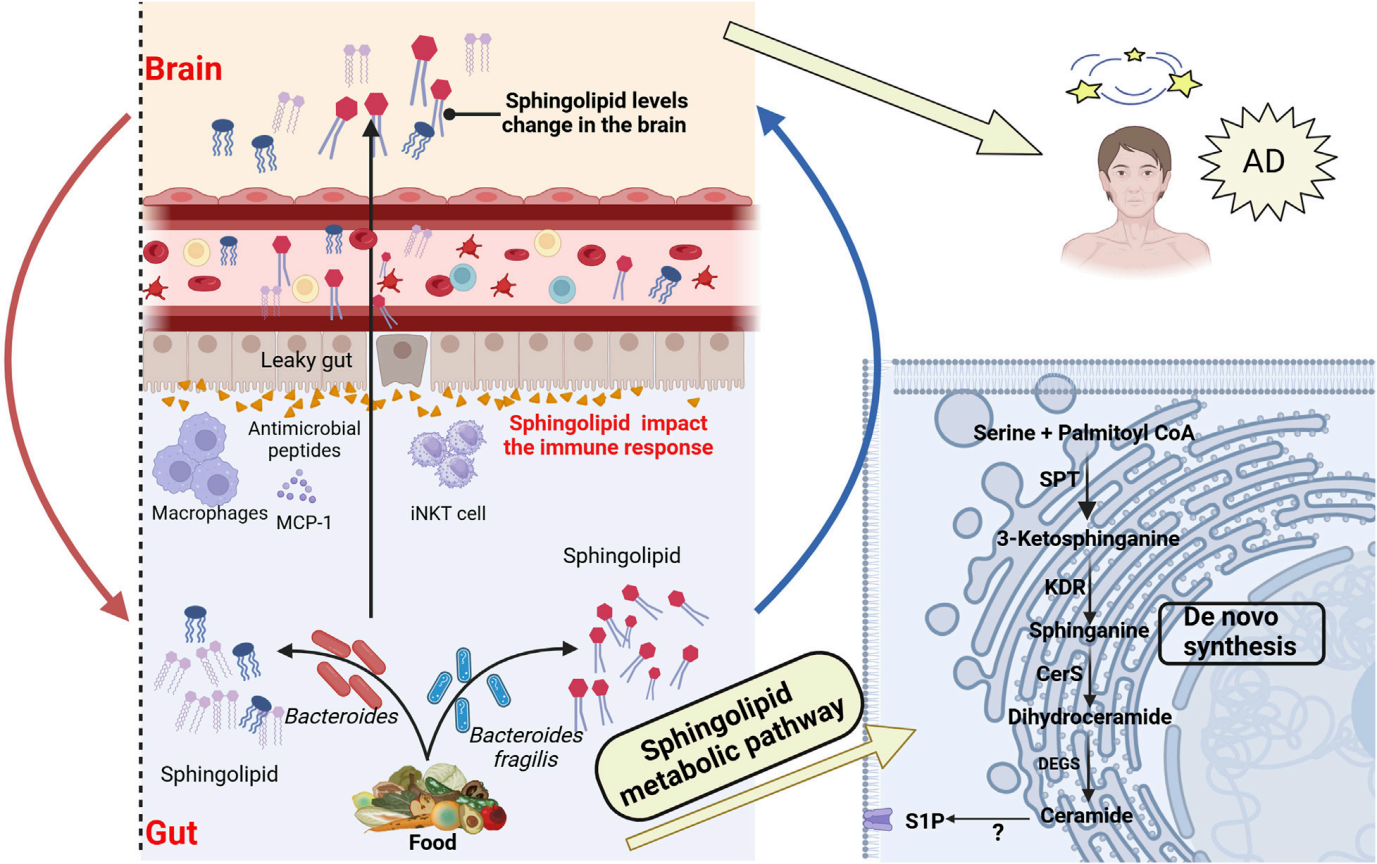
Association between bacterial sphingolipid metabolism and AD. Intestinal bacterial sphingolipids, as endogenous sources, catalyze the generation of ceramides via the *de novo* pathway. This process involves serine-palmitoyltransferase (SPT) in the endoplasmic reticulum, after which the ceramides are transferred to the Golgi apparatus. Bacterial sphingolipids, in conjunction with dietary sphingolipids, play a crucial role in modulating host sphingolipid homeostasis. Bacterial sphingolipids indirectly affect the pathogenesis of AD by modulating the activity of various immune system components. This includes invariant natural killer T (iNKT) cells, macrophages, and antimicrobial peptides, all of which are integral to the immune response. Moreover, Bacterial sphingolipids may mediate the progression of AD through several mechanisms. They are involved in regulating Aβ aggregation, Tau protein phosphorylation, abnormal microglial activation, and neuronal necrosis. These processes are critical factors in the development and progression of AD. SPT, Serine palmitoyltransferase; KDR, 3-Keto-dihydrosphingosine reductase; Cers, Ceramide synthase; DEGS, Dihydroceramide desaturase; S1P, Sphingosine 1-phosphate. Created with (biorender.com).

Sphingolipids are ubiquitously present in eukaryotes such as plants and animals. With advancements in chemical and lipid technologies, it has been discovered that a minority of bacteria possess enzymatic capabilities to synthesize sphingolipids (Bai et al., 2023). Bacterial phyla known to produce sphingolipids include *Bacteroidetes*, *α-Proteobacteria*, and *δ-Proteobacteria* (Brown et al., 2023). Within the gut microbiota, *Bacteroidetes* is currently the only known symbiotic bacteria capable of synthesizing sphingolipids. These bacteria use serine-palmitoyl transferase to catalyze the *de novo* synthesis of sphingolipids (Le et al., 2022; Heaver et al., 2018) and play a crucial role in inositol sphingolipid synthesis (Heaver et al., 2022). Especially in the absence of dietary intake, gut bacteria serve as a source of endogenous sphingolipids. Johnson’s research supported this notion, showing that in mice on a sphingolipid-deficient diet, intestinal epithelial cells absorb long-chain sphingoid bases (Sa(d18:0) and Sa(d17:0)) from the lumen and metabolize them via *de novo* synthesis pathways (Johnson et al., 2020), as described in Figure 3. Bacterial and dietary sphingolipids interact to maintain stable sphingolipid levels in the host (Rohrhofer et al., 2021), further highlighting the potential role of bacterial sphingolipids in the gut-brain axis.

Sphingolipids, which include ceramide, sphingosine-1-phosphate (S1P), and sphingosine, are bioactive substances commonly found in cell membranes. In particular, they are abundant in central nervous system tissues (van Echten-Deckert and Walter, 2012). Studies have shown that sphingolipids play a central role in cell signaling and regulation of homeostasis, and are crucial in processes like mitochondrial apoptosis, autophagy, immune regulation, and oxidative stress (Brown et al., 2023). These functions position sphingolipids as key players in the pathogenesis of various diseases, with AD drawing significant attention. In patients with AD, cerebrospinal fluid and blood samples have shown that blood sphingolipid concentrations are associated with brain atrophy and cognitive decline (Varma et al., 2018). A longitudinal cohort study also found increased expression of sphingolipid S1P 18:1 (Chua et al., 2020), which may induce the upregulation of astrocytes and various inflammatory mediators, suggesting that sphingolipids may be early biomarkers for AD, as shown in Figure 3. Moreover, experimental results from animal models further support this conclusion (Crivelli et al., 2021). As summarized by Luo (Luo et al., 2024), ceramide contributes to the stability of BACE1 and promotes excessive production of Aβ (Parveen et al., 2019). Additionally, neutral sphingomyelinase inhibitors can reduce the release of ceramide vesicles, inhibit Aβ aggregation, and improve cognitive function (Dinkins et al., 2017). Interestingly, acidic sphingomyelinase ceramide inhibitors can also reduce the release of ceramide and pro-inflammatory factors from microglia (Crivelli et al., 2023). Additionally, in Tg2576 AD model mice, decreasing the accumulation of ganglioside GM3 has been shown to lower soluble Aβ42 levels and amyloid plaque burden, thereby stabilizing remote memory (Dodge et al., 2022). Sulfatide deficiency can activate microglia, increase the expression of AD risk genes such as Apoe, Trem2, Cd33, and Mmp12, and exacerbate cognitive impairment in mouse models. These findings are consistent with recent research indicating that impaired sphingolipid metabolism is associated with the development of AD (Qiu et al., 2021). However, given that sphingolipids are considered early biomarkers of AD, further investigation is required to elucidate the relationship and underlying mechanisms between sphingolipids and AD. This research may offer potential strategies to delay the onset and progression of the disease.

Zhong and colleagues discovered that the activation of S1P transport protein is related to microglial inflammation and NF-κB pathway activation (Zhong et al., 2019). Additionally, the binding of S1P to S1PR1 mediates the secretion of pro-inflammatory cytokines and chemokines, increasing central nervous system inflammation (O’Sullivan et al., 2018). Kong’s research further found that ceramide promotes the binding of tubulin to voltage-dependent anion channel 1, further blocking mitochondrial energy metabolism and increasing the risk of AD (Kong et al., 2018). Additionally, the metabolic regulation of ceramides is closely linked to autophagy, mTORC1 activity, and Aβ secretion, further exacerbating the progression of AD pathology (Ordóñez-Gutiérrez et al., 2018). However, most research has focused on exogenous dietary sphingolipids, while the importance of endogenous sphingolipids for gut health has been largely overlooked. Interestingly, Brown and colleagues discovered that a deficiency in endogenous sphingolipids derived from *Bacteroides* can lead to intestinal mucosal damage, intestinal inflammation, and alterations in the host ceramide pool (Brown EM. et al., 2019). Multiple reports have indicated that *Bacteroides* sphingolipids influence the gut-brain axis through immune regulation, lipid metabolism, and other pathways. For example, inositol derived from *Bacteroidetes* may affect the secretion of host antimicrobial peptides, thereby participating in host immune defense (Heaver et al., 2022). Additionally, gut bacteria, as a source of endogenous sphingolipids, can increase the host’s available sphingolipid pool, thereby influencing host sphingolipid metabolism (Johnson et al., 2020). Further studies reveal that *Bacteroides*-derived sphingolipids are associated with MCP-1 secretion and macrophage release (Brown EM. et al., 2019). Moreover, *Bacteroides fragilis* can alter the homeostasis of host iNKT cells, thereby inhibiting the proliferation of iNKT cells in the colon and protecting the integrity of intestinal cells and mucosa (An et al., 2014). These findings reveal the multiple mechanisms by which gut microbiota-derived sphingolipids function within the gut-brain axis, suggesting that endogenous sphingolipids may hold potential value in the prevention and treatment of neurodegenerative diseases (Le et al., 2022; Wei et al., 2009).

Interestingly, the study found that hawthorn flavonoids increase levels of sphingolipids and phosphatidylcholine, which can reverse gut microbiota dysbiosis, inhibit Aβ accumulation, and suppress abnormal microglial activation in the hippocampus (Zhang J. et al., 2022). In addition, glycerophospho dihydroceramide derived from *Porphyromonas gingivalis* has been found to promote Aβ formation and Tau hyperphosphorylation *in vitro*. This process is also accompanied by an increase in senescence-associated secretory phenotype factors, and downregulates Sirt-1 expression. These may be potential mechanisms for the development of AD (Yamada et al., 2020). Targeted metabolomics studies have further confirmed that disruptions in sphingolipid metabolism are closely associated with the biological changes observed in the preclinical and prodromal stages of AD, particularly in relation to Tau phosphorylation, Aβ metabolism, and apoptosis (Varma et al., 2018). These data suggest that bacterial sphingolipids can regulate host sphingolipid synthesis and immune homeostasis, indirectly influencing the onset of AD, as illustrated in Figure 3. However, current research on gut microbiota-derived sphingolipids in AD patients is not yet comprehensive. It is anticipated that this field will soon emerge as a novel target for AD treatment.

#### 4.1.2 Phospholipids

As the role of bacterial sphingolipids in the pathogenesis of AD is gradually being revealed, phospholipids, another critical class of lipids, are also recognized for their significant role in the gut-brain axis and neurodegenerative disorders. Phospholipids, as major components of cell membranes, maintain membrane stability and fluidity, and provide an optimal environment for protein interactions and transport (Bohdanowicz and Grinstein, 2013). The brain, being the organ with the highest lipid content, exhibits significant alterations in lipid composition in AD (Svennerholm, 1968). High-performance liquid chromatography analysis revealed a reduction of approximately 20% in total phospholipids in the frontal cortex and hippocampus of AD patients, when normalized to DNA content (Guan et al., 1999). This disruption in phospholipid levels may be closely related to the pathogenesis of AD.

As early as 2001, Folch extracts from autopsy materials revealed significantly reduced levels of phosphatidylethanolamine (PE) and phosphatidylinositol in the brains of AD patients (Pettegrew et al., 2001). Changes in these phospholipids may influence the progression of AD through the gut-brain axis. Phospholipids synthesized by gut microbiota, particularly PE, could exacerbate this pathological process by disrupting the host’s phospholipid metabolism (Brown et al., 2023). For instance, researchers have screened and identified an immunologically active molecule produced by *Akkermansia muciniphila*: a diacyl phosphatidylethanolamine (a15:0-i15:0 PE) with two branched chains. This molecule can activate the immune system, induce the release of certain inflammatory factors, compromise the intestinal mucosal barrier, and trigger various immune diseases, including AD (Li Y. et al., 2022).

Despite this, the pathways through which phospholipids interact between the host and symbionts remain largely unknown. On the other hand, studies indicated that hypoxic conditions lead to increased intestinal angiogenin-4 secretion, resulting in a significant increase in *Desulfovibrio* abundance in mice intestines. *Desulfovibrio* species produce PE and phosphatidylcholine (Li Y. et al., 2022). These two phospholipids are major components of brain phospholipids (Blusztajn and Slack, 2023). The excessive breakdown of phosphatidylcholine and PE is considered one of the most prominent metabolic defects in AD. Their reduced concentrations are crucial factors in mitochondrial dysfunction, Aβ accumulation, and memory deficits (Zappelli et al., 2022). Additionally, recent studies have revealed the significant role of α-glycerophosphoethanolamine in protecting aging hippocampal neurons. It protects astrocytes by increasing glucose uptake, reducing total and oligomeric α-synuclein, and decreasing total tau accumulation (Daniele et al., 2020). Multi-omics analyses reveal that disruptions in glycerophospholipid metabolism lead to alterations in gut microbiota, potentially serving as a primary mechanism for heightened microglial activation and neuroinflammatory responses in APP/PS1 mice. This pathway is critically implicated in the pathogenesis and progression of AD (Qian et al., 2023). Reports indicate that phosphatidylcholine rich in DHA and EPA improves memory and cognitive function in APP/PS1 mice by inhibiting Aβ production, reducing oxidative stress, and decreasing apoptosis (Che et al., 2018). In summary, disruptions in phospholipid metabolism, particularly involving PE and phosphatidylcholine, may contribute to the onset and progression of AD through various mechanisms. Future research should explore these metabolic pathways to develop more effective AD treatment strategies.

#### 4.1.3 Lipopolysaccharide

Building on the relationship between phospholipid metabolism and AD, microbiota-derived LPS are also key factors influencing the progression of AD. LPS is currently the only glycolipid metabolite produced by the microbiome identified (Pahil et al., 2024). LPS is a potent endotoxin primarily found in Gram-negative bacteria, such as *Bacteroides fragilis*, *Escherichia coli*, and *Shigella flexneri* (Günther et al., 2020; Khalid et al., 2019). Zhao et al., (2017) first reported the presence of bacterial LPS in the hippocampus and temporal lobe cortex of AD patients, with levels in the hippocampus of late-stage AD patients being 26 times higher than in age-matched controls. This finding provides important insights into the relationship between LPS and the pathogenesis of AD. Similar results were observed in AD model mice further support this perspective. Interestingly, intermittent feeding with high doses of LPS can also cause learning and memory impairments and hippocampal neuronal damage in mice (Engler-Chiurazzi et al., 2023). These results indicated that the high expression of LPS in the blood and brain induced by gut infections may be a high-risk factor for the onset and progression of AD.

Further studies have shown that LPS affects various AD-related pathological products, including Aβ homeostasis, tau pathology, neuroinflammation, and neurodegeneration (Molinaro et al., 2020; Kim et al., 2021). Mice administered LPS exhibit a decline in memory performance, which may be related to increased expression of glial fibrillary acidic protein and p-Tau protein in the hippocampal CA3 region, along with elevated levels of pro-inflammatory factors and oxidative responses (Azzam et al., 2023). More importantly, studies on PgLPS transgenic mice have further elucidated the relationship between LPS and the AD phenotype. The study found that PgLPS transgenic mice induce AD phenotypes, including increased expression of pro-inflammatory factors, Tau hyperphosphorylation (Jiang M. et al., 2021), enhanced microglia-mediated neuroinflammation, and intracellular Aβ accumulation in neurons (Wu et al., 2017). These results reveal the multifaceted role of LPS in exacerbating AD pathology.

Furthermore, LPS-induced innate immune responses and neuroinflammation are crucial in AD pathogenesis. Luo’s research found that LPS is associated with microglial activation and neuroinflammation (Luo et al., 2024). LPS regulates the inflammatory response of microglia by modulating the expression of Toll-like receptor 4 and triggering receptor expressed on myeloid cells 2, thereby affecting Aβ clearance (Zhu et al., 2022; Zhou et al., 2019). At the same time, a non-targeted metabolomics study found that LPS can regulate energy metabolism in the hippocampus of APP/PS1 mice, promote pro-inflammatory activation of microglia (Agostini et al., 2020). Importantly, TLR4 can induce “Aβ/LPS tolerance,” increasing M1 polarization of microglia, which leads to reduced Aβ clearance and the expression of AD risk genes (Yang et al., 2021). Additionally, building on Luo’s research, it has been found that LPS can activate the chemotactic response of astrocytes, leading to increased inflammatory infiltration by neutrophils and T cells (Lopez-Rodriguez et al., 2021; Hennessy et al., 2015). In addition to inducing microglial and astrocyte activation, which increases AD risk, LPS can cross the gut barrier into the bloodstream, disrupt the BBB, and increase the release of pro-inflammatory factors (Brown and Heneka, 2024). This process suggests that LPS may drive the progression of AD by promoting inflammation and dysregulation of the immune system.

Notably, LPS can also directly induce apoptosis and necrosis in neural cells and neurons (Seim et al., 2019). Research indicated that colonization by *Aeromonas* and elevated LPS levels cause hippocampal neuronal apoptosis and spatial memory impairments in mice (Wang X. et al., 2023). Further studies revealed that exposure to LPS significantly increased the protein and mRNA expression levels of G protein-coupled receptor 17 (GPR17) in the hippocampus. GPR17 may contribute to neuronal damage and cognitive impairments by regulating the NF-κB p65/CREB/BDNF(cAMP response element-binding protein/brain-derived neurotrophic factor) signaling pathway, thus promoting the expression of oxidative stress and inflammatory factors (Liang et al., 2023). Additionally, the impact of LPS on myelination should not be overlooked.The overall level of myelination in the cortex and hippocampus of APP/PS1 mice, as well as *postmortem* AD tissue, is reduced. LPS can also affect the remyelination of axons (Regen et al., 2011; Dean et al., 2017), and age-dependent structural defects in myelin directly and indirectly promote the formation of Aβ plaques (Depp et al., 2023). As discussed by Luo and colleagues, LPS may become a new target for future AD research (Luo et al., 2024).

#### 4.1.4 Short-chain fatty acids

As another class of lipids produced by gut microbiota metabolism, SCFAs have a significant impact on the central nervous system, playing an essential role in maintaining the integrity of the BBB and regulating neuroinflammation (Hoyles et al., 2018; Liu J. et al., 2020). SCFAs, such as acetate, propionate, butyrate, and valerate, are lipids produced by the gut microbiome, such as *Bacteroides*, *Firmicutes*, and *Akkermansia muciniphila*, through the fermentation of dietary fiber (Lynch and Pedersen, 2016; Dalile et al., 2019). These SCFAs serve not only as an energy source for intestinal epithelial cells but also influence systemic organs, including the liver and brain, through the bloodstream (Qian et al., 2022; Zhou Y. et al., 2023). Oldendorf and coworkers found in 1973 that acetate, propionate, and butyrate, which are SCFAs, are able to pass through the BBB in rats after being injected with labeled SCFAs (Oldendorf, 1973). This finding further reveals the potential role of SCFAs in the pathogenesis of AD. SCFAs are indispensable for establishing a normal BBB and maintaining its integrity. Therefore, it is crucial to further explore the role of SCFAs in the BBB integrity.

Research has found that germ-free mice exhibit increased BBB permeability; however, gut microbes producing SCFAs reduce this permeability by upregulating the expression of tight junction proteins, indicating the role of SCFAs in maintaining cerebral homeostasis (Hoyles et al., 2018; Xie et al., 2023a). Further research found that propionate (at physiological concentration of 1 μM) inhibits pathways related to nonspecific microbial infections through a CD14-dependent mechanism, reduces LRP-1 expression, and protects the BBB against oxidative stress via the Nuclear factor erythroid 2-related factor 2 signaling pathway (Hoyles et al., 2018). The reduction in LRP1 levels can significantly inhibit the uptake and spread of Tau protein in neurons, helping to slow AD progression (Rauch et al., 2020). Conversely, broad-spectrum antibiotic treatment and germ-free mice both exhibit significantly increased barrier permeability and disrupted tight junctions between endothelial cells. Increasing butyrate intake can reverse this barrier disruption (Wen et al., 2020). Evidence suggests that histone acetylation is a key epigenetic mechanism in AD development (Yang et al., 2017). SCFAs may maintain BBB integrity through pathways mediated by GPR and the inhibition of histone deacetylases (Fock and Parnova, 2023; Matt et al., 2018).

Current research suggest that the primary pathogenic mechanisms of AD are Aβ deposition and abnormal phosphorylation of Tau protein, leading to neuroinflammation and neurodegenerative changes. In the feces of patients with mild cognitive impairment, propionate and butyrate are negatively correlated with Aβ42 (Nagpal et al., 2019). Clinical studies have shown that Aβ levels in AD patients are positively correlated with serum acetate and pentanoate levels, and negatively correlated with butyrate levels (Marizzoni et al., 2020). This suggests that SCFAs play a critical role in neuroprotection and Aβ metabolism, potentially providing protective effects by interfering with the assembly and conversion mechanisms of Aβ peptides (Ho et al., 2018). Subsequent studies have found that sodium butyrate can upregulate the PI3K/AKT/CREB/BDNF signaling pathway, inhibit the overactivation of microglia and Aβ deposition, and promote long-term potentiation and synaptic plasticity (Jiang Y. et al., 2021). Interestingly, other studies have shown that SCFAs can upregulate apolipoprotein E, increasing microglial activation and Aβ plaque recruitment (Colombo et al., 2021). These differing results may be due to variations in SCFAs concentrations and metabolite imbalances, leading to different outcomes in each study.

In neuroinflammation, microglia and astrocytes, as the brain’s innate immune cell populations, play a crucial role (Kwon and Koh, 2020). Recent studies indicated that SCFAs modulate the maturation of microglia and synaptic plasticity, thereby affecting learning, memory, and behavior. Erny et al. reported that mice lacking receptors for SCFAs exhibited microglial defects under germ-free conditions (Erny et al., 2021). Previous reports have found that acetate exerts anti-inflammatory effects by upregulating GPR41 levels in BV2 cells and inhibiting NF-κB (Luo et al., 2024; Liu J. et al., 2020). Importantly, gut-derived SCFAs, such as acetate, can regulate microglial functions in the 5xFAD AD mouse model, including mitochondrial metabolism and phagocytic function (Erny et al., 2021). Butyrate can also inhibit the secretion of pro-inflammatory cytokines by microglia, ameliorating related neuroinflammation (Matt et al., 2018). Astrocytes also play a crucial role in the central nervous system, regulating neurotransmitter secretion, synaptic function, and BBB integrity, thus maintaining central nervous system homeostasis.

A potential mechanism by which the MGBA affects behavior and cognition is through myelination in the prefrontal cortex (Ntranos and Casaccia, 2018). The PFC integrates inputs from multiple brain regions, processing complex decision-making and memory retrieval. SCFAs can alleviate behavioral and cognitive impairments by improving myelin abnormalities. For example, antibiotic treatment in neonatal mice can lead to oligodendrocyte damage and alterations in PFC myelin phospholipids, accompanied by behavioral deficits and gut dysbiosis, which can be prevented by SCFAs supplementation (Keogh et al., 2021). Butyrate can directly promote the differentiation and maturation of oligodendrocytes, inhibit demyelination, and enhance remyelination (Chen et al., 2019). In summary, SCFAs regulate brain function through various mechanisms, including maintaining BBB integrity, modulating glial cell activation, inhibiting demyelination, inhibiting Aβ deposition, and enhancing synaptic plasticity, as described in Table 1. Although the specific mechanisms of SCFAs remain controversial, their role as key mediators in the MGBA is widely recognized and is expected to be further elucidated in future research.

### 4.2 Bile acids

Following the discussion on the impact of SCFAs on AD, bile acids (BAs), another important gut metabolite, also exhibit complex regulatory roles in the pathogenesis of AD. BAs are important metabolic and immune signaling molecules. Primary bile acids, such as cholic acid and chenodeoxycholic acid, are synthesized in hepatocytes and secreted into the intestine, where they are metabolized by gut bacteria such as *Bifidobacterium* and *Bacteroides* into secondary bile acids, including lithocholic acid and deoxycholic acid (Luo et al., 2024). BAs enter the brain through both active transport and passive diffusion. In rat brains, 20 types of BAs have been identified, including 9 unconjugated BAs and 11 conjugated BAs (Zheng et al., 2016).

BAs and the gut microbiome have a close bidirectional regulation. They control host metabolism by interacting with the nuclear farnesoid X receptor and G protein-coupled receptor 5 (Collins et al., 2023). The microbiome can modify and transform bile acids through processes such as 7α-dehydroxylation, oxidation, desulfation, esterification, epimerization, and deconjugation (Hou et al., 2022). This interaction sets the groundwork for researching the MGBA’s control over host metabolism (Wang S. et al., 2023). The transport of BAs between the gut and the brain may underlie the involvement of gut microbiota in AD pathogenesis. A multi-omics study found that the concentrations of 7α-hydroxycholesterol, cholic acid, and chenodeoxycholic acid in the serum of AD patients are associated with brain amyloid deposition and brain atrophy (Varma et al., 2021). Additionally, an association between serum bile acid profiles and cerebrospinal fluid biomarkers in AD patients, such as amyloid and tau proteins, has been identified. However, these findings still require further validation through clinical studies and animal models (Nho et al., 2019). A large multicenter study found that the concentration of cholic acid in the serum of AD patients was significantly reduced, while the levels of bacterially produced secondary bile acids, such as deoxycholic acid, were increased. This may be related to changes in bacterial 7β-dehydroxylase activity, leading to increased levels of neurotoxic bile acids (MahmoudianDehkordi et al., 2019).

Some bile acids have been reported to mediate neuroprotective effects in AD patients. Preclinical studies have shown that ursodeoxycholic acid and tauroursodeoxycholic acid protect mitochondrial function and improve neuropsychiatric symptoms by reducing oxidative stress, decreasing cell apoptosis, and inhibiting the production of pro-inflammatory factors such as TNF-α and IL-1β (Huang et al., 2022). The neuroprotective effects of tauroursodeoxycholic acid were confirmed early in animal experiments (Lo et al., 2013; Nunes et al., 2012). A series of animal model studies found that tauroursodeoxycholic acid improves cognitive and behavioral abnormalities in transgenic mice by enhancing glucose metabolism (Zangerolamo et al., 2021), reducing endoplasmic reticulum stress (Ochiai et al., 2021), inhibiting Aβ formation, and preventing Tau hyperphosphorylation (Song et al., 2024). Additionally, ursodeoxycholic acid can reduce apoptotic cascade reactions (West et al., 2020), increase mitochondrial membrane potential, and enhance mitochondrial respiration (Bell et al., 2018). These effects help improve mitochondrial function and mitigate AD pathology.

Studies have found that the apical sodium-bile acid transporter enhances intestinal bile acid absorption, leading to the accumulation of conjugated primary bile acids and ammonia in the brain. This accumulation is positively correlated with hippocampal neuron loss, synaptic damage, and cognitive impairment, which may be the reason for increased neurotoxic bile acids in AD patients (Ren et al., 2024). However, the mechanisms by which bile acids contribute to the pathogenesis of AD are not yet fully understood and may involve the FXR/TGR5/GLP-1 pathway and the FXR/FGF15/19 pathway (Chen C. et al., 2023; Yusta et al., 2017). BAs directly activate FXR on intestinal cells, inducing the release of GLP-1. In the central nervous system, GLP-1, acting as a signaling molecule, inhibits the NF-κB signaling pathway and decreases IL-1β and TNF-αmRNA expression in LPS-stimulated microglia, thus modulating brain metabolism (Lee et al., 2018). Bile acids stimulate the secretion of FGF15/19 by activating FXR in ileal cells. FGF-19 can cross the BBB and bind to fibroblast growth factor receptors in specific brain areas (Ren et al., 2022), such as the hypothalamus and the dorsal vagal complex, thereby modulating brain function. Based on these studies, future research should further explore the impact of these pathways on the pathogenesis of AD.

### 4.3 Amino acids

Amino acids, as crucial nutrients, are increasingly evidenced to regulate central nervous system functions via the gut-brain axis. In this process, functional amino acids are crucial for maintaining gut homeostasis (Wikoff et al., 2009). Particularly, aromatic amino acids with unique aromatic ring structures, like tyrosine and tryptophan, play key roles in neurotransmitter synthesis, neuroinflammatory responses, and cognitive function (Zhao et al., 2022).

First, tyrosine serves as a precursor to catecholamine neurotransmitters like dopamine, norepinephrine, and epinephrine. Research has found that defects in tubulin tyrosination cause synaptic function and plasticity deficits, which are critical for signal transduction dysfunction during AD pathology (Peris et al., 2022). Interestingly, protein tyrosine phosphatases, as regulators of neural signal transduction, are abnormally expressed in AD patients and are associated with abnormal Aβ and Tau accumulation and synaptic damage (Zhao et al., 2022). Additionally, spleen tyrosine kinase is essential for maintaining the aggregation of microglia around Aβ plaques (Schweig et al., 2017). Therefore, the regulatory role of tyrosine in cognitive memory associated with AD should not be overlooked.

Similarly, tryptophan, as a key precursor in neurotransmitter synthesis (Boadle-Biber, 1993), has metabolites that are also of significant interest for their impact on AD pathology. Tryptophan, one of the nine essential amino acids, is primarily obtained from the diet. Gut microbiome can break down tryptophan to produce various metabolites (Wikoff et al., 2009). For instance, *Clostridium sporogenes* produce the serotonin, while *Escherichia coli* and *Lactobacillus gasseri* convert it into indole. A double-blind crossover clinical trial on tryptophan depletion found that acute tryptophan depletion severely impaired cognitive function in AD patients (Porter et al., 2000). Consequently, tryptophan has gradually gained attention as a key mediator in the MGBA influencing AD. The tryptophan metabolism pathway primarily involves serotonin, indole, and kynurenic acid (Roth et al., 2021). The kynurenic acid pathway is the foremost metabolic route for tryptophan, through which over 90% of tryptophan is degraded into various bioactive compounds. Compelling evidence suggest that the kynurenine pathway is associated with behavioral and cognitive symptoms in neurological diseases (Fu et al., 2023). As early as the 1990s, studies confirmed that kynurenic acid levels and the plasma kynurenic acid/tryptophan ratio are releated to the clinical characteristics and adverse outcomes of different diseases (Saito et al., 1993; Heyes et al., 1993), involving AD. The inflammatory process in AD is characterized by the shift of tryptophan metabolism from the serotonin pathway to the neuroinflammatory kynurenine pathway (Whiley et al., 2021). A multicenter cohort study found that a higher kynurenine/tryptophan ratio is associated with gray matter atrophy, amyloid deposition, and total Tau accumulation. Therefore, increased kynurenine metabolism may be a key factor in inducing the inflammatory signaling cascade in AD (Willette et al., 2021). Recent studies have found a positive correlation between kynurenine levels in cerebrospinal fluid and Aβ1-42 (Bakker et al., 2023). However, previous research have shown a negative correlation between plasma kynurenine and cerebrospinal fluid p-tau (Jacobs et al., 2019). This discrepancy may be related to the imbalance between the neurotoxic quinolinic acid and the neuroprotective kynurenic acid in the kynurenine pathway. Quinolinic acid, a downstream metabolite of the kynurenine pathway, is a neurotoxin associated with the pathogenesis of AD (Guillemin and Brew, 2002). It affects dendritic spine regeneration, reduces BDNF levels, and induces cognitive impairment by disrupting the cytoskeleton, activating oxidative stress (Chen et al., 2021; Ting et al., 2009), and targeting reactive astrocytes. Conversely, an increase in the neuroprotective kynurenic acid may be a reason for the slow progression of AD (Knapskog et al., 2023).

In addition, indole substances produced in the tryptophan metabolic pathway are activators in AhR signaling process. Reports indicate that indolepropionic acid, as an AhR agonist, can activate the immune system, reduce oxidative damage, inhibit lipid accumulation, and improve cognitive impairment (Niu et al., 2024). Studies have shown that tryptophan deficiency in male APP/PS1 mice leads to an imbalance in indole-producing bacteria, which can induce behavioral and cognitive defects in mice (Sun J. et al., 2022). Research has found that the neuroprotective effects of indole are mediated through the activation of the AhR pathway. This activation inhibits inflammasomes and the production of pro-inflammatory factors, prevents Aβ accumulation and tau hyperphosphorylation, and restores synaptic plasticity (Sun J. et al., 2022). Interestingly, a high-tryptophan diet can inhibit microglial M1 polarization, reduce the release of pro-inflammatory factors, and decrease Aβ load in neurons of transgenic animal models. This effect may be related to the activation of the AhR pathway and the regulation of gut microbiota dysbiosis (Pan et al., 2024; Noristani et al., 2012). In transgenic model mice, AhR activation has been found to improve cognitive impairment and deficits by upregulating Aβ degradation. This evidence suggests that neuroinflammation in AD patients may be related to decreased AhR expression. With the advent of technologies such as tryptophan nanoparticles (Sharma et al., 2022), tryptophan is gradually becoming a potential therapeutic target for AD. Although the mechanisms of AhR in AD are complex, research suggests that it may serve as a novel therapeutic target for future AD treatments.

Similar to the metabolic roles of tyrosine and tryptophan, glutamate, as an excitatory neurotransmitter, plays a critical role in maintaining neurotransmitter balance in AD. It is converted into the inhibitory neurotransmitter GABA through the bacterial glutamate decarboxylase system (Zott and Konnerth, 2023). Thus, Alterations in glutamate metabolism apparently impact neural function in AD. Glutamate levels in the cerebrospinal fluid of AD patients are elevated (Pomara et al., 1992), but *postmortem* brain tissue has shown reduced glutamate content (Chalmers et al., 1990). This may be related to dysfunction in glutamate metabolism and dysregulation of glutamate kinetics (Brymer et al., 2023). Furthermore, the dysbiosis of gut microbiota in AD patients impacts glutamate metabolism. Enhancers of N-methyl-D-aspartate glutamate receptors have been shown to ameliorate cognitive impairment in AD patients (Chang et al., 2020). In summary, amino acids such as tyrosine, tryptophan, and glutamate collectively influence the pathological progression of AD through complex metabolic pathways and neurotransmitter regulation mechanisms. These amino acids not only play crucial roles in the synthesis and metabolism of neurotransmitters but also become key factors in AD pathology by regulating neuroinflammation and oxidative stress.

### 4.4 Neurotransmitters and gut microbiome

Amino acids serve as the building blocks for neurotransmitter synthesis, and gut microbiota further influence the synthesis and function of neurotransmitters by regulating these amino acids (Whiley et al., 2021; Zott and Konnerth, 2023). Extensive research have shown that the gut microbiome can synthesize and regulate neuroactive substances in the host, such as GABA, 5-hydroxytryptamine (5-HT), dopamine, and norepinephrine (Wikoff et al., 2009; Chang et al., 2020). For instance, mice raised in germ-free conditions exhibit significantly reduced levels of serotonin and dopamine (Nishino et al., 2013), due to the absence of microbial-host interactions, highlighting the crucial role of gut microbiota in neurotransmitter synthesis. Furthermore, human commensal bacteria, such as *Brevilactibacter* and *Bifidobacterium dentium*, have been demonstrated to produce GABA (Barrett et al., 2012). Colonization by Roseburia increases levels of serotonin (5-HT) (Zhou M. et al., 2023). Therefore, neuroactive substances produced by microbes can affect neuronal metabolism and brain function through the MGBA, further supporting the bidirectional communication mechanism between gut microbiota and the central nervous system.

#### 4.4.1 GABA

GABA, the central nervous system’s predominant inhibitory neurotransmitter (Lee et al., 2019), has the excitatory neurotransmitter glutamate as its metabolic precursor. It has been reported that an imbalance in GABA metabolism can lead to cognitive and memory impairments, and is associated with anxiety, depression, and AD (Govindpani et al., 2020). Autopsy findings reveal a downregulation of GABA signaling components in the middle temporal gyrus of AD patients, accompanied by reduced GABA levels in the cerebrospinal fluid (Carello-Collar et al., 2023). Numerous animal studies have found that GABA is primarily produced by intestinal microorganisms such as *Lacticigenium*, *Bacteroides*, *Bifidobacterium*, and *Escherichia coli*. This finding has been corroborated by human fecal transcriptome analysis (Chang et al., 2020; Strandwitz et al., 2019). Additionally, studies have found that germ-free mice have lower GABA levels in their colon (Eicher and Mohajeri, 2022). These findings support the view that gut microbiota can regulate host GABA levels. However, the connection and mechanism of action between gut GABA and the brain still need to be completed.

Although dietary glutamate cannot cross the BBB, GABA produced by bacterial metabolism can enter the brain and regulate neuronal and synaptic changes (Zhang H. et al., 2022). A recent human study found that fecal microbiome transplantation alters plasma GABA levels (Fu et al., 2023). Moreover, *Bifidobacterium dentium* was found to secrete GABA and acetate *in vitro* experiments (Engevik et al., 2021), leading to increased levels of GABA and tyrosine in mouse feces and brain (Luck et al., 2021). Thus, the gut microbiome may indirectly regulate GABA signaling to enhance brain function and behavior. In both the presynaptic and postsynaptic stages of brain transmission, GABA binds to particular transmembrane receptors on the cell membrane. Furthermore, it is involved in the proliferation of precursor neurons, the formation of synapses, and the suppression of inflammation (Tang X. et al., 2021). Similarly, the reduction of GABA_B_ receptors on glial cells can induce the upregulation of Aβ1-40 in transgenic mice (Osse et al., 2023). Animal research indicated that GABA can regulate IL-15 expression in the hippocampus, potentially impairing the new object recognition ability in mice (Di Castro et al., 2024). Additionally, research indicates that astrocytes play a critical role in the GABA metabolic cycle. Insufficient astrocyte metabolism leads to reduced glutamine synthesis, which in turn inhibits neuronal GABA synthesis, resulting in an imbalance between synaptic excitation and inhibition in the AD brain (Andersen et al., 2021).

Bi et al. (2020) suggested that dysfunction in the GABAergic system leads to neuronal excitatory/inhibitory imbalance, which in turn promotes the spread of Aβ and Tau pathology, exacerbating cognitive impairment in AD patients. Thus, abnormalities in the GABAergic system might represent a common target in the various aberrant signaling pathways of AD. Increasing research have revealed the critical role of GABA activity in cognitive functions such as learning and motor coordination (Wen et al., 2022b). Studies indicated that GABA activation promotes neuronal differentiation (Tozuka et al., 2005). However, other research showed that inhibiting GABA signaling can alleviate cognitive impairment in AD mice (Hardy et al., 1987). In the brains of late-stage AD patients, although the glutamate and GABA receptor subunits are relatively unaffected, these findings suggest that the brain continues to attempt to maintain the balance of excitatory and inhibitory tensions even in the final stages of the disease (Carello-Collar et al., 2023). Consequently, a new concept has emerged: the balance between inhibitory and excitatory expression of GABA may be crucial in preventing the onset of AD (Bi et al., 2020).

Unfortunately, fluctuations in GABA levels in the AD brain have been primarily confirmed through animal and post-mortem studies. Consistent observations include increased GABA levels in the hippocampus and astrocytes of AD model mice, along with an increase in amyloid protein deposition (Jo et al., 2014; Rice et al., 2019). Therefore, further exploration of the GABAergic system may become a key target for restoring excitatory/inhibitory balance and cognitive function in AD patients.

#### 4.4.2 5-hydroxytryptamine

Gut microbes synthesize 90% of the body’s 5-HT (Boadle-Biber, 1993), for instance, *Lacticigenium* and *Bifidobacterium* (Tian et al., 2022), using tryptophan from dietary proteins within the gastrointestinal epithelial cells (Martin et al., 2017). Importantly, 5-HT cannot cross the BBB (Pagire et al., 2022), preventing it from directly influencing brain 5-HT levels. Intriguingly, research by Wang indicated that *Clostridium butyricum* can alleviate learning and memory impairments induced by Di-(2-ethylhexyl) Phthalate, attributed to the restoration of gut microbiome, suppression of inflammatory factors, and increased 5-HT secretion (Wang et al., 2023d). Consequently, the role of gut microbiota and 5-HT in regulating central nervous system function is gaining increasing attention.

The study found that T*PH2 KO CUMS* mice (chronic mild stress with serotonin inhibition), there was a significant decrease in extracellular serotonin levels, accompanied by gut microbiome dysbiosis and aggravated cognitive dysfunction. Feeding with *Lacticigenium* E001-B-8 significantly improved the cognitive behavior of these mice (Ma et al., 2023). Moreover, selective serotonin reuptake inhibitors (SSRIs), a class of antidepressants, increase extracellular serotonin levels by inhibiting the reuptake of the neurotransmitter serotonin by nerve synapses (Michely et al., 2020). Studies have found that citalopram improves cognition in APP mice by regulating mitochondrial biogenesis and autophagy. Additionally, it has been shown to increase dendritic spine density and synaptic activity in these mice (Reddy et al., 2021). Cellular experiments show similar results. Additionally, *in vitro* cell experiments have shown that citalopram reduces p-Tau and serotonin protein expression, improves mitochondrial respiration, and increases cell survival rates (Sawant et al., 2024). However, recent clinicopathological studies show that SSRIs accelerate cognitive decline by increasing Tau tangles (Sood et al., 2023). Additionally, there is evidence that increased serotonin synthesis is associated with temporal lobe atrophy (Markova et al., 2023). These findings highlight the crucial role of the gut microbiome in the central nervous system’s 5-HT signaling pathway.

Although the role of gut microbiota-derived 5-HT in the central nervous system is gaining increasing attention, the mechanisms underlying their connection remain unclear. Tryptophan, the precursor of 5-HT, influences the brain through the kynurenine and indole pathways (Cervenka et al., 2017). Additionally, 5-HT released from enterochromaffin cells in the gut signals to intestinal neurons via the Trapa1 pathway (Ye et al., 2021), enhancing vagal sensory neuron activity. Further research has found that gastric distension stimulate enterochromaffin cells to release 5-HT, which then activates 5-HT3 receptors on the gastric vagal nerve. It ultimately activates neurons in the paraventricular and supraoptic nuclie by increasing c-fos expression in the tractus solitarius and area postrema (Leon et al., 2021; Mazda et al., 2004). Importantly, the coordination of 5-HT receptor function is crucial for cognition and memory in AD patients. Abnormal serotonergic signaling has been observed in the hippocampus of AD model mice, further supporting the significant role of 5-HT in AD (Wang et al., 2023e).

More importantly, activation of 5-HT7R increases Tau phosphorylation and aggregation via CDK5, leading to neuronal death, long-term potentiation deficits, and memory impairment in transgenic mice (Labus et al., 2021; Ackmann et al., 2024). Wu also confirmed similar results, showing that the downregulation of 5-HT1AR and 5-HT2AR can promote neuronal resistance to Aβ neurotoxicity and improve cognitive deficits (Wu et al., 2024). Indeed, variations in the gut microbiome can influence the levels of tryptophan and serotonin, which in turn may influence the synthesis of crucial brain neurotransmitters (Doifode et al., 2021; Cryan et al., 2019). These findings collectively support the significant role of the interaction between 5-HT signaling and gut microbiota in the pathogenesis of AD. However, the lack of precise 5-HT detection methods has significantly slowed the progress in research on 5-HT signaling (Wan et al., 2021).

#### 4.4.3 Other neurotransmitters

Acetylcholine, a common excitatory neurotransmitter, is prevalent in both the peripheral and central nervous system (Picciotto et al., 2012). In recent years, research have revealed a connection between gut microbiota and the central nervous system, particularly noting that various gut microbes release acetylcholine, such as *Lactiplantibacillus plantarum*, *Escherichia coli*, *Bacillus subtilis*, and *Staphylococcus aureus* (Horiuchi et al., 2003). Since acetylcholine cannot directly cross the BBB (Amenta et al., 2001), choline from the peripheral nervous system crosses the BBB with the help of carrier proteins and is then synthesized into acetylcholine in the central nervous system by choline acetyltransferase (Inazu, 2019). Studies have found reduced acetylcholine transferase activity across all cortical regions in AD patients (Rossor et al., 1982), suggesting that acetylcholine levels in the memory-related prefrontal cortex may be a potential factor influencing the development of AD (Sun Q. et al., 2022). Further research indicates that impairments in cholinergic neurotransmission are closely linked to the pathological process of AD. For example, disruptions in cortical cholinergic neurotransmission can affect the expression and processing of amyloid precursor protein amyloid precursor protein (APP), leading to the deposition of Aβ amyloid plaques, which in turn further disrupt cholinergic transmission (Schliebs, 2005). This series of studies was confirmed in another animal experiment. In AD model mice, obstruction of pyramidal neuron pathways in the prefrontal cortex and cholinergic neuron pathways in the basal forebrain is observed, which is associated with cognitive decline (Zhong et al., 2022). Clinical studies indicated that the basal forebrain cholinergic system can predict Aβ deposition in AD (Berry and Harrison, 2023), which positively correlates with the extent of hippocampal atrophy (Teipel et al., 2023).

Like acetylcholine, dopamine plays various roles in the central nervous system, but its mechanisms of action in AD may differ. Dopamine is a crucial neurotransmitter in the vertebrate system and a catecholamine that affect both the central and peripheral nervous systems (Miri et al., 2023). Interestingly, more than half of dopamine is produced in the gut, with *Bacillus*, *Bacteroides*, *Brevilactibacter*, and *Bifidobacterium* being the major contributors to its production and bioavailability (Eisenhofer et al., 1997). Dopamine metabolized by the microbiome is transported to the brain across the BBB, where it negatively regulates the NLRP3 inflammasome via dopamine receptors expressed in microglia and astrocytes, thereby influencing neuroinflammatory responses (Possemato et al., 2023). The dopamine D4 receptor modulates the transport of α-amino-3-hydroxy-5-methyl-4-isoxazole-propionic acid receptor in the GABAergic interneurons of the prefrontal cortex through a unique signaling mechanism, regulating the strength of excitatory synapses in neurons and affecting cognition and emotion (Penn et al., 2017; Valles-Colomer et al., 2019). In AD model rats, degeneration of midbrain limbic dopamine neurons impairs the function of parvalbumin interneurons (La Barbera et al., 2022), leading to early hippocampal hyperexcitability and epilepsy-like activity (Spoleti et al., 2024), which contrasts with the role of acetylcholine in AD. Additionally, Calabresi et al. highlight that dopamine not only affects mood and cognition but also regulates behavior (Korn et al., 2021), learning, and memory (Calabresi et al., 2007).

Similar to dopamine, norepinephrine is another catecholamine produced by gut bacteria (Dicks, 2022). Although present in small amounts, it significantly affects human cognition and behavior and is an important neurotransmitter (Borodovitsyna et al., 2017). While primarily synthesized in the adrenal medulla, norepinephrine is also produced by gut bacteria such as *Escherichia coli*, *Proteus vulgaris*, and *Bacillus subtilis* (Ojeda et al., 2021). Reports indicated that norepinephrine can modulate synaptic plasticity (Fukabori et al., 2020), regulate microglial and astrocytic activation, and increase the production of BDNF, thereby providing neuroprotection (Giustino et al., 2020). These functions are crucial for maintaining brain health, particularly in the context of neurodegenerative diseases like AD. *Postmortem* cerebrospinal fluid samples from AD patients show reduced levels of norepinephrine and its metabolites (Kaddurah-Daouk et al., 2011), which may serve as evidence of norepinephrine’s involvement in the pathogenesis of AD. Studies in *APP/PS1* transgenic mice showed that neuroinflammation and microglial activation are significantly associated with degeneration in the locus coeruleus-norepinephrine system (Cao et al., 2021). Additionally, deficiencies in this system are associated with Aβ deposition (JaMalatt and Tagliati, 2022), hyperphosphorylation of Tau protein (Chalermpalanupap et al., 2018), and spatial memory deficits (Betts et al., 2019; Lin et al., 2024). Locus coeruleus-norepinephrine may play a crucial role in the pathology of AD by reducing inflammatory cytokine release and enhancing amyloid clearance through the regulation of microglial migration and phagocytic functions (Flores-Aguilar et al., 2022; Li J. et al., 2022).

The gut microbiome also produce various other neurotransmitters, including histamine (produced by *Enterococci*) (De Palma et al., 2022) and tyramine (produced by *Enterococcus*) (O’Donnell et al., 2020). These neurotransmitters are hypothesized to regulate Aβ aggregation, Tau protein accumulation, and synaptic remodeling via the gut-brain axis, affecting brain physiology (Berry and Harrison, 2023; Fukabori et al., 2020; JaMalatt and Tagliati, 2022),. However, their functions have not been fully elucidated and represent a potential direction for future research. As our understanding of these complex interactions deepens, we hope to develop new therapeutic strategies to more effectively address AD, a global health challenge.

## 5 Therapeutic strategies targeting gut microbiota modulation for AD

**FIGURE 4:**
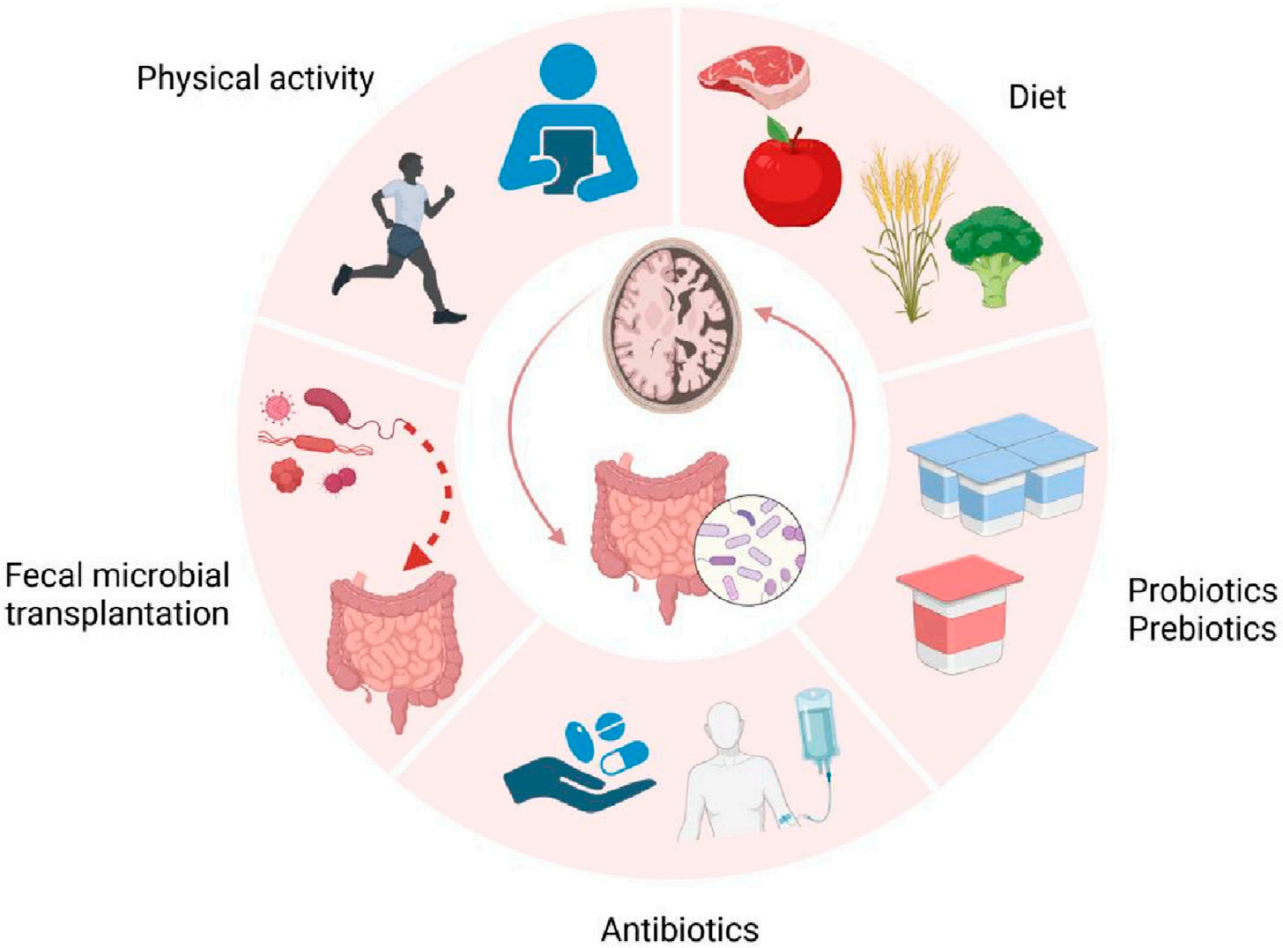
Gut-brain axis-related AD treatment strategies. Environmental factors such as diet, sleep, and exercise contribute to the onset and progression of AD. Conversely, healthy lifestyle habits like proper nutrition and physical activity, along with probiotics/prebiotics, antibiotics and gut microbiota transplantation, may serve as potential treatment approaches for AD. Created with (biorender.com).

As discussed, gut microbiome metabolites regulate AD through various mechanisms, including Aβ deposition, Tau protein phosphorylation, synaptic damage, and BBB permeability. However unhealthy dietary habits, lack of exercise, and poor sleep patterns increase the risk of developing AD (Seo et al., 2019; Seo and Holtzman, 2024). Currently, methods for modulating the gut microbiome in AD patients are being explored, including lifestyle changes, probiotics and prebiotics, antibiotic therapy, fecal microbiome transplantation, as described in Figure 4. Intestinal microbial communities can be restored ecologically through these interventions, offering potential new targets for AD treatment.

### 5.1 Lifestyle and gut microbiome

AD-related lifestyle impairments have become a global concern. To date, no specific medication can cure AD or significantly reverse its progression. The link between lifestyle factors, such as diet and exercise, and AD has been widely confirmed. Epidemiological evidences indicated that various dietary patterns, exercise regimens, and sleep habits are associated with AD risk (Ma et al., 2020; Liu YH. et al., 2020; Jayedi et al., 2020; van den Brink et al., 2019), as described in Figure 4.

Research indicated that different dietary patterns play varying roles in the pathology and cognitive function of AD (Zhang et al., 2020; Dissanayaka et al., 2024; Dietrich et al., 2022). The Mediterranean diet, rich in fiber and quality fats, modulates cerebrospinal fluid biomarkers (like Aβ40, Aβ42, and T-Tau), reverses neuroinflammation, and alters gut and BBB permeability (Hoscheidt et al., 2022). In contrast, the Western diet, high in saturated fats, exacerbates AD progression by altering lipid genes, impairing barrier functions, and reducing cerebral perfusion (Hoscheidt et al., 2022). Evidence suggested that diet may influence Aβ production and tau protein processing, or it may modulate neuroinflammation, metabolism, and oxidative stress related to AD, all of which could be associated with the gut microbiome and its metabolites (Ayten and Bilici, 2024). A ketogenic diet, high in fats and low in carbohydrates, may interfere with Aβ aggregation and Tau protein phosphorylation, thereby altering brain function (Mazandarani et al., 2023). Studies have shown that patients with mild cognitive impairment, a modified Mediterranean ketogenic diet results in changes in gut bacterial phyla, families, and genera, with an increase in beneficial SCFAs, including butyrate (Nagpal et al., 2019; Hoscheidt et al., 2022; Lee et al., 2022; Gregory et al., 2023). Interestingly, dietary fiber can interact with intestinal epithelial and immune cells and modulate gut barrier and immune functions through the AMPK/TLR pathway, reducing gut permeability (Cai et al., 2020; Nakamura et al., 2023). Shi and colleagues found that mice fed a fiber-deficient diet exhibited a reduction in *Bacteroidetes* and *Proteus*, impaired gut barrier function accompanied by decreased SCFAs production (Shi et al., 2021). This also affected the ultrastructure and function of synapses in the hippocampal region, leading to cognitive deficits such as spatial memory and daily living skills.

Beyond the impact of specific dietary patterns, dietary fiber plays a crucial role in modulating gut microbiota and maintaining gut barrier function. Additionally, intermittent fasting, as another dietary intervention, influences neuronal survival and synaptic function by altering the composition of gut microbiota (Cignarella et al., 2018), thereby improving behavioral abnormalities and cognitive impairment (Yang Q. et al., 2023). Notably, the practice of Bigu, originating in the pre-Qin period, is known for its health and longevity benefits (Tang L. et al., 2021). However, its therapeutic effects on cognitive functions and the underlying mechanisms have not been systematically elucidated. Pan found that IF can inhibit carbohydrate metabolism, increase amino acid abundance, alleviate Aβ burden and excessively activate neuroglial cells (Pan et al., 2022). Another similar study found that IF reduces intestinal mucosal permeability, alters microbial metabolites, suppresses neuroinflammatory responses, and thus preserves synaptic ultrastructure, mitigating cognitive and spatial memory impairments (Liu Z. et al., 2020). This may be closely related to mitochondrial biogenesis and energy metabolism gene expression in the hippocampus. In summary, dietary patterns are significant modulators of metabolic functions, cerebrovascular health, AD pathology, and cognitive functions, with different dietary compositions affecting the gut microbiome and its metabolites (Guo M. et al., 2023). Extensive experimental and clinical research is required to determine the optimal diet, which could be an effective intervention to prevent the onset of AD or slow its progression.

As our understanding of dietary interventions deepens, research indicates that other lifestyle factors, particularly physical activity, may also influence the progression of AD [(Adjetey et al., 2023; Bracco et al., 2023; Khairy and Salama, 2023; Brown BM. et al., 2019). Physical activity may not only be associated with Aβ and Tau protein levels but also improve learning and memory decline by reducing superoxide dismutase 9 (Guo C. et al., 2023), promoting cholesterol homeostasis (Hu et al., 2023), and decreasing neuronal death and apoptosis (Feng et al., 2023). Another animal research found that aerobic exercise enhances synaptic growth and prevents synaptic damage, improving spatial learning and memory in *APP/PS1* mice. This improvement may be related to the modulation of the GPR81/cAMP/PKA signaling pathway and the reversal of microglial cell phenotypes (Yang J. et al., 2023). Recent research suggest that another potential mechanism through which exercise affects cognition is by modulating the gut microbiome and its metabolites (Peng et al., 2024). Yuan found that long-term treadmill exercise alleviated oxidative stress in APP/PS1 transgenic AD mice (Yuan et al., 2024), protected the intestinal barrier, and reduced brain LPS accumulation through the combined action of the gut-liver-Kupffer cell axis. Human studies also indicate that exercise can prevent age-related cognitive decline and reduce the risk of AD-related dementia (Sandison et al., 2023). A prospective randomized controlled trial showed that a multimodal lifestyle intervention, including diet and exercise, effectively improved cognitive decline and was more effective than pharmacological interventions (Roach et al., 2023). The neurobiological and pathological mechanisms linking exercise and cognition have been extensively studied. Delgado-Peraza discovered that exercise increases the levels of neuroprotective factors (including BDNF, proBDNF, and humanin) in neuron-derived extracellular vesicles (Delgado-Peraza et al., 2023), particularly in AD subjects carrying the *APOEε4* allele. A cross-sectional study involving 4,322 patients showed a positive correlation between physical activity and hippocampal brain volume (Aslanyan et al., 2023). In summary, these findings support physical activity as an effective strategy to reduce or prevent AD. The mechanisms involve regulating the production of bioactive substances, reducing oxidative stress and inflammation, enhancing BDNF signaling, improving hippocampal neurogenesis and mitochondrial function, increasing cerebral blood flow, and decreasing AD-related pathology (Veronese et al., 2019; Chandra et al., 2023b; Tarawneh and Penhos, 2022; Andrade-Guerrero et al., 2023).

Moreover, AD patients often exhibit disrupted sleep rhythms, insufficient sleep duration, and fragmented sleep, indicating that sleep could be a potential intervention target for AD (Knopman et al., 2021). In summary, diet, exercise, and sleep, as potential interventions for AD, not only individually impact the gut-brain axis but may also synergistically enhance neuroprotection through their interactions. Future large-scale studies should focus on exploring the combined effects of these lifestyle modifications to identify optimal strategies for preventing the onset or delaying the progression of AD.

### 5.2 Probiotics and prebiotics

In addition to lifestyle modifications, probiotics and their derivatives have increasingly become a focal point in AD treatment research in recent years. Antibiotics, known for their bactericidal and bacteriostatic properties, are not suitable for routine treatment of AD due to safety and specificity limitations. Given this, probiotics and prebiotics, which influence the gut-brain axis via the gut microbiome, are considered potential therapeutic agents for AD (Ansari et al., 2023). Given the incomplete understanding of the complex relationship between microbiome and AD, the potential benefits of probiotics and prebiotics are still at the preliminary stages of research. However, animal experiments and clinical studies have already demonstrated their crucial role in AD, as described in Figure 4.

Probiotics are beneficial live microorganisms, typically *Bifidobacterium* (di Vito et al., 2023), that aim to restore gut ecological balance, reduce the release of toxic substances, and promote neuronal and axonal repair through various mechanisms, as described in Table 2. Increasing evidence suggests that probiotics not only improve gut health but also influence brain function and cognitive abilities through the gut-brain axis (Den et al., 2020). Recent studies on AD animal models have revealed the potential benefits of probiotics in mitigating AD-related pathological manifestations. For example, probiotics can enhance intestinal barrier permeability and reduce the release of toxic metabolites and bacteria into the bloodstream, and consequently alleviate inflammation in the central nervous system (di Vito et al., 2023; Sichetti et al., 2018). More specifically, probiotic formulations comprising *Lacticaseibacillus rhamnosus*, *Bifidobacterium lactis*, and *Bifidobacterium longum* enhance anti-inflammatory responses by inducing the production of cytokines such as IL-10, increasing the M2 macrophage phenotype, and improving the anti-inflammatory properties of the intestinal epithelial barrier (di Vito et al., 2023; Sichetti et al., 2018). Additionally, it has been demonstrated that probiotics can modulate gut microbiome composition (Toscano et al., 2017) and the microbiota-gut-brain axis. Specifically, ProBiotic-4 improves neuronal and synaptic damage, modulates glial activation, and alters microbiota composition by inhibiting TLR4 and RIG-I-mediated NF-κB signaling pathways and inflammatory responses (Yang et al., 2020). Through this mechanism, probiotics not only enhance gut barrier function but also inhibit inflammation-induced gut-brain axis dysregulation, thereby alleviating pathological changes in AD mouse models (Yadav et al., 2023).

**TABLE 2:** Effects of probiotic/prebiotic supplementation on gut microbiota and brain function.

Probiotic/Prebiotic Type	Probiotic/Prebiotic	Effect on Brain Function	Ref.
Probiotic	*Lacticaseibacillus rhamnosus, Bifidobacterium lactis, Bifidobacterium longum*	Increase macrophage M2 phenotype and anti-inflammatory cytokine IL-10 secretion, inhibit the release of pro-inflammatory cytokine IL-6, and improve intestinal epithelial barrier	di Vito et al. (2023), Sichetti et al. (2018)
	*Lactobacillus rhamnosus, Bifidobacterium longum*	Reduce potentially harmful bacteria and increase beneficial bacteria	Toscano et al. (2017)
	SLAB51 (multi-strain probiotic)	Modulate lipid metabolism, restore glucose homeostasis, reduce Aβ plaque accumulation and oxidative stress in AD models, and improve cognitive levels	Bonfili et al. (2017), Bonfili et al. (2022), Bonfili et al. (2020), Bonfili et al. (2018)
	ProBiotic-4	Inhibit TLR4/RIG-I/NF-κB signaling pathway, improve neuronal and synaptic damage, and regulate neural activity	Yang et al. (2020)
	Nisin	Reduce pro-inflammatory cytokine mRNA expression and reduce Aβ42 and P-Tau deposition	Zhao et al. (2023)
Prebiotic	Mannan-oligosaccharides	Remodel the intestinal microbiome, protect the intestinal barrier, and enhance the formation of SCFAs	Liu et al. (2021)
	Prebiotic R13	Inhibit C/EBPβ/AEP signaling pathway, reduce intestinal leakage and oxidative stress, and restore cognitive function	Chen et al. (2020)
	Yeast β-glucan	Modulate gut microbiota and promote short-chain fatty acid production, improve neuroinflammation and insulin resistance	Xu et al. (2020)
	Fructooligosaccharides	Modulate microbiota composition, improve neuroinflammation via TLR4-Myd88-NF-κB pathway	Sun et al. (2019), Zhang et al. (2023)

Further research has revealed that probiotics can directly influence pathological features associated with AD, as shown in Table 2. A study reported that oral supplementation with the multi-strain probiotic formulation SLAB51 reduced Aβ plaque accumulation and brain damage in the triple transgenic mouse model of AD (*3xTg-AD*) (Bonfili et al., 2017). Recent findings suggested that Nisin can reduce deposits of Aβ42, total Tau, and phosphorylated Tau, thereby ameliorating periodontitis-induced dysbiosis of the brain microbiome and Alzheimer’s-like neuroinflammation (Zhao et al., 2023). The effects of probiotics are not limited to inflammation modulation and the reduction of pathological features; they also extend to the regulation of energy metabolism. Furthermore, probiotics can improve the fatty acid profile in mice, reduce the ω-6/ω-3 ratio, and decrease cholesterol synthesis (Bonfili et al., 2022). Additionally, probiotics increase the key glucose transporters (GLUT3 and GLUT1) expression in the brain and the insulin-like growth factor receptor β, reduce the phosphorylation of protein kinase B, and improve the mechanisms of impaired glucose metabolism in AD, thereby slowing disease progression (Bonfili et al., 2020; Bonfili et al., 2018). Overall, an increasing number of studies have confirmed the potential value of probiotic therapy in AD, particularly in modulating gut microbiota, reducing AD-related pathological substances, promoting the production of neuroprotective factors, and facilitating neural repair (Nguyen et al., 2023).

In addition to probiotics, prebiotics have also shown potential roles in AD therapy (Ni Lochlainn et al., 2024). As an energy source for probiotics, prebiotics can stimulate the growth and proliferation of beneficial bacteria (Roberfroid et al., 2010). Animals models of neurodegenerative diseases have also demonstrated that prebiotics improved behavioral and cognitive impairments as well as reduced intestinal inflammation, as depicted in Table 2. It has been reported that mannan-oligosaccharides can reshape the gut microbiome, protect the integrity of the intestinal barrier, and enhance the formation of neuroprotective metabolites, such as SCFAs (Zha et al., 2023). Researchers have also found that mannan-oligosaccharides can inhibit neuroinflammatory responses, regulate disturbances in the HPA axis, and reduce the accumulation of Aβ in the cerebral cortex, hippocampus, and amygdala, likely mediated by SCFAs (Liu et al., 2021). Similarly, Chen et al. discovered that prebiotic R13 induces Ligilactobacillus salivarius to antagonize the CCAAT/enhancer-binding protein β/asparagine endopeptidase (C/EBPβ/AEP) pathway, mitigating intestinal leakage and oxidative stress, and restoring behavioral and cognitive functions in 3xTg mice (Chen et al., 2020). Additionally, yeast β-glucan can not only modulate gut microbiota and promote the production of SCFAs but also improve neuroinflammation and insulin resistance, providing significant protective effects in the early stages of AD (Xu et al., 2020). Beyond animal studies, the preventive and therapeutic effects of prebiotics on AD have also been validated in several clinical studies. A large longitudinal study in elderly individuals have shown that higher dietary intake of fructan reduces the risk of AD (Nishikawa et al., 2021). Recent studies have also found that low fructooligosaccharides not only regulate gut microbiota composition but also alleviate neuroinflammation through the TLR4-Myd88-NF-κB pathway (Sun et al., 2019; Zhang et al., 2023). In conclusion, probiotics and prebiotics have shown broad potential in the treatment of AD by modulating gut microbiota, as shown in Table 2. Although existing research results are promising, larger clinical trials are needed to further validate their long-term efficacy and safety, particularly concerning dosage optimization and strain selection.

### 5.3 Antibiotics

In addition to lifestyle and probiotic treatment strategies, antibiotics are gradually emerging as a new direction in AD therapy research. Numerous studies indicated that infections with pathogens, such as herpes simplex virus type 1, adenovirus, *Chlamydia* pneumonia, and *Porphyromonas gingivalis*, may be among the factors contributing to the onset of AD (Yamada et al., 2020; Hegde et al., 2019; Gérard et al., 2005; Zhao et al., 2024). Consequently, the exploration of antibiotics and antiviral drugs as a potential therapeutic approach is gaining momentum (Lin et al., 2013; Iqbal et al., 2020).

The mechanism of action of pathogens may involve various aspects, including the activation of the host’s immune response, disruption of the gut-brain barrier, activation of glial cells, and resulting in neuronal and axonal damage (Zhao et al., 2024; Honjo et al., 2009; Xie et al., 2023b). Therefore, antibiotics and antiviral drugs may offer a solution to this predicament (Angelucci et al., 2019), as described in Figure 4. Interestingly, an epidemiological research reported this possibility. Rakuša (Rakuša et al., 2023) and colleagues analyzed public health insurance data from Germany to investigate the association between sporadic antibiotic use and the risk of dementia. The results indicated that previous antibiotic use might reduce the risk of dementia by decreasing inflammation. Similar effects have been observed in animal studies (Cheng et al., 2023). Furthermore, Wang (Wang et al., 2019) discovered that an antibiotic solution containing ampicillin, streptomycin, and vancomycin could block Th1 cell infiltration and M1 microglial cell activation in AD mouse models. It was also proposed that gut dysbiosis promotes Th1 cell infiltration, leading to local crosstalk with M1 microglial cells and subsequently triggering the differentiation of microglia into a pro-inflammatory state (Schetters et al., 2018). Furthermore, rifampicin mediates anti-inflammatory effects, inhibits Tau protein hyperphosphorylation, counters amyloid protein formation, and enhances cholinergic function, thereby protecting neurons and improving cognition (Yulug et al., 2018).

Notably, antiviral drugs have also shown potential efficacy in the treatment of AD. Low-dose efavirenz, as an antiretroviral medication, enhances CYP46A1 activity, thereby promoting brain cholesterol metabolism in patients with early Alzheimer’s disease (Lerner et al., 2022). Similar results have also been observed in AD mouse models (Petrov et al., 2019). Additionally, the enhancement of CYP46A1 activity can reduce amyloid plaque burden in the cerebral cortex and subcortical regions, inhibit microglial activation, lower Aβ levels, and decrease mortality (Mast et al., 2017). During the early stages of HIV infection, CCR5-tropic viruses dominate. The CCR5 antagonist Maraviroc not only reduces viral load in the blood and brain but also enhances Aβ efflux and trans-endothelial transport via the LRP1 pathway, thereby protecting neurons and improving cognition (Bhargavan et al., 2021). Furthermore, a retrospective cohort study found that treating with anti-herpetic drugs can reduce the risk of dementia induced by herpes virus (Tzeng et al., 2018). Additionally, research has shown that interferon can reverse hippocampal inflammation and cognitive impairments in rats caused by Aβ1-42, while also reducing oxidative stress markers such as ROS and lipid peroxidation (Mudò et al., 2019).

In addition, antibiotics can not only control bacterial infections but also modulate the gut microbiota (Minter et al., 2016), indirectly influencing central nervous system pathology, reducing Aβ plaque deposition, and inhibiting excessive microglial activation (Zou et al., 2023; Dodiya et al., 2020; Dodiya et al., 2019; Bosch et al., 2024). Although antibiotics have shown therapeutic potential, their non-specific effects can lead to potential side effects (Zarrinpar et al., 2018), particularly in disrupting the balance of the gut microbiome. For instance, mice fed with antibiotics exhibit gut microbiome dysbiosis, such as a decline in the abundance of butyrate-producing *Bacteroides S24-7* and short-chain fatty acid-producing *Bacteroides*, leading to cognitive deficits and behavioral abnormalities (Tirelle et al., 2020). Additionally, there is a reduction in the levels of BDNF in the hippocampal region of mice, exacerbating learning and memory impairments (Zeraati et al., 2019). Therefore, the use of antibiotics as a potential future intervention for AD presents certain challenges. In fact, antiviral drugs are currently being investigated in double-blind, placebo-controlled trials in AD patients (Panza et al., 2019). However, due to the limitations related to the diversity, non-specificity, standardization, and resistance associated with antibiotics, further research and optimization are necessary.

### 5.4 Fecal microbial transplantation

Due to the non-specific effects of antibiotics and their disruptive impact on gut microbiota, researchers have begun exploring fecal microbiota transplantation (FMT) as an emerging alternative therapy. FMT involves transferring the fecal microbiome from a healthy donor to the gastrointestinal tract of the recipien, first used to treat recurrent *Clostridium difficile* infection (Juul et al., 2018; BBagdasarian et al., 2015; Lee et al., 2016). With the advancement of research in the MGBA, FMT has shown positive effects on AD (Biazzo and Deidda, 2022; Park et al., 2022; Xiang et al., 2023), as described in Figure 4. Studies have shown that FMT can reduce brain amyloid pathology and Tau protein phosphorylation, thereby ameliorating neurodegenerative changes associated with AD (Biazzo and Deidda, 2022; Kim et al., 2020). Advances in genomics have shown that *ADL* ^*PAPT*^ mice exhibit loss of epithelial barrier integrity and chronic intestinal and systemic inflammation (Kim et al., 2020). Following gut microbiome transplantation from wild-type mice, improvements were observed in brain tissue with reduced Aβ plaques and neurofibrillary tangles, along with enhanced mice reactivity and cognitive function. This also reversed gastrointestinal inflammation and the gene expression of inflammatory cells in the blood (Kim et al., 2020; Behling et al., 2024). Furthermore, FMT can restore the imbalance of the gut microbiome in mice with traumatic brain injury, improving ventricular enlargement and behavioral abnormalities (Davis et al., 2022).

With advancements in microbiological techniques and genomics, researchers have further isolated dominant gut microbiota from healthy intestines (Mitsuoka, 1970). Research have suggested that dominant gut microbiome can modulate pro-inflammatory cytokines, APP expression; they also reduce the area of Aβ plaque deposits in the brain’s hippocampus, restoring gut microbial balance (Li M. et al., 2023). This approach not only overcomes the limitations of probiotics and traditional FMT but also demonstrates significant potential in the treatment of AD. Future research will further validate the clinical safety and efficacy of FMT, with the potential to mitigate neurodegenerative changes in AD patients by modulating the gut-brain axis. However, the widespread application of FMT still faces challenges related to ethics, efficacy, and safety.

## 6 Conclusion

In recent years, a growing body of research has highlighted the critical role of gut microbiota and their metabolites in the pathogenesis of AD. In this review, we comprehensively explore the multiple roles of gut microbial metabolites in AD, revealing how these metabolites influence the complex processes of neurodegeneration through the MGBA. Current research demonstrates that metabolites such as lipids, amino acids, and bile acids play pivotal roles in the pathogenesis and progression of AD. Specifically, they are critical in the regulation of Aβ metabolism and Tau protein phosphorylation, and may serve as key biomarkers for early diagnosis and monitoring of AD. However, despite the increasing understanding of these metabolites, their precise effects at different stages of AD remain unclear, presenting significant opportunities for future research.

Compared to previous studies, this paper further elucidates the potential of microbial metabolites in regulating various pathogenic mechanisms via the MGBA. For instance, SCFAs have the potential to directly impact the central nervous system by maintaining BBB integrity, inhibiting neuroinflammation, and enhancing synaptic plasticity, demonstrating their potential in AD therapy. Additionally, BAs regulate neuroprotection and metabolic processes through pathways such as FXR and TGR5. However, their impact on AD remains incompletely understood, warranting further exploration of these molecular mechanisms in future studies. However, the current research presents significant limitations. Firstly, although significant progress has been made in animal and *in vitro* studies, there is a lack of large-scale, systematic human clinical studies. The considerable variability in gut microbiota across individuals may result in low comparability between different study outcomes. Secondly, existing studies predominantly focus on the effects of individual metabolites, often overlooking the potential complex interactions between these metabolites. Therefore, future research should place greater emphasis on the dynamic interactions between microbial metabolites and the host nervous system, exploring their specific roles across different pathological stages.

Although research is still evolving, gut microbial metabolites have demonstrated significant potential as therapeutic targets for AD. Based on current research, we propose an innovative strategy for treating AD: long-term dietary and lifestyle adjustments, combined with probiotics, antibiotics, or gut microbiota transplantation, aimed at restoring the balance of the gut microbiome and metabolites. However, translating these research findings into clinical applications requires overcoming several challenges, including elucidating the specific mechanisms of gut microbial metabolites in AD, optimizing intervention strategies, and ensuring the safety and efficacy of clinical applications. With the ongoing advancements in metagenomics, metabolomics, and systems biology, we are likely to further unravel the complex mechanisms of the gut-brain axis and develop more precise and effective therapeutic approaches.
